# Waist circumference as a parameter in school-based interventions to prevent overweight and obesity - a systematic review and meta-analysis

**DOI:** 10.1186/s12889-024-20354-7

**Published:** 2024-10-17

**Authors:** Antje Kula, Ricarda Brender, Kerstin Melissa Bernartz, Ulla Walter

**Affiliations:** 1 Institute for Epidemiology, Social Medicine and Health System Research, Hannover Medical School, Carl-Neuberg-Str. 1, D-30625 Hannover, Germany; 2 Medical School OWL, Bielefeld University, Postfach 10 01 31, D-33501 Bielefeld, Germany

**Keywords:** Childhood obesity, Abdominal obesity, Prevention, Waist circumference, Systematic review, Meta-analysis

## Abstract

**Background:**

Preventing childhood obesity remains an important public health issue worldwide. Since visceral fat in particular is understood as an important risk factor for many chronic diseases, waist circumference is recommended as a measurement parameter for global obesity surveillance. This systematic review and meta-analysis focused on waist circumference as an outcome parameter for studies of school-based interventions to prevent overweight and obesity.

**Methods:**

A systematic literature search was conducted at the end of 2019 in nine data bases, including Medline and Embase, in order to identify relevant studies evaluating interventions in schools aimed at preventing obesity. Eligibility criteria admitted randomised and non-randomised controlled trials. After screening titles, abstracts and full texts, the data of the identified studies were systematically extracted. Risk of bias was assessed according to study type with the appropriate Cochrane Risk of Bias Tool. The review gives a qualitative overview over all included studies structured by extracted data. Separate meta-analyses were done for the outcome mean difference in change in waist circumference, measured in cm or reported as z-score value, using an inverse variance random-effects model due to study design.

**Results:**

A total of 2421 publications were screened based on titles, abstracts and full texts. Complemented by results of a former systematic literature search 44 studies were identified for inclusion, comprising a total of 39.837 participants (age range: 6 to 18 years). Nearly half of the studies were conducted in Europe, two-thirds combined diet and exercise-based interventions. Likewise two thirds of the studies were conducted as cluster-randomised trials. Most of the reported effects favoured the experimental groups, indicating the basic effectiveness of school-based measures. Based on reported data, only one third of the studies could be included in the meta-analyses. For the difference in mean change of the outcome parameter waist circumference measured in cm (95% CI), we found a pooled effect estimate of -0.95 (-1.87; -0.46). For the difference in mean change of the outcome parameter waist circumference reported as z-score value (95% CI), the pooled effect estimate was -0.10 (-0.15; -0.05). Both effect estimates were in favour of the experimental group. The overall effect sizes were small with a p-value < 0.05.

**Conclusions:**

Pooled effect estimates were small but in favour of the experimental groups. The same applies to the majority of the effects reported in the included studies. The included cluster of randomised controlled trials demonstrated an especially sound methodological standard. The possibility of achieving larger effects in studies of preventive interventions and health promotion is limited. Schools can only realise their full potential in preventing overweight and obesity in children and adolescents if they are accompanied by measures in other areas of the obesogenic environment.

**Supplementary Information:**

The online version contains supplementary material available at 10.1186/s12889-024-20354-7.

## Background

The prevalence of overweight and obesity in children has increased worldwide over the last four decades [1, 2]. Although there are indications that the increase has at least stopped in some countries, prevalence rates are still high [3].

According to the World Health Organisation (WHO) overweight is a condition of excessive fat deposits and obesity is a chronic complex disease defined by excessive fat deposits [4]. Higher morbidity and mortality rates due to overweight and obesity have been proven in many studies [5]. This serious threat and restriction of individual health also leads to an overburdening of care systems and high financial costs [6, 7].

Childhood and juvenile overweight and obesity have an impact on current health and are associated with a range of chronic diseases in adulthood [8–10]. Therefore early prevention of obesity remains a key area for action worldwide. Schools are in a unique position to reach nearly all children and adolescents to promote healthy behaviors [11, 12]. School-based interventions are seen as a key component in preventing overweight and obesity in childhood and youth [13]. A systematic review and meta-analysis evaluating school-based obesity prevention interventions reported a pooled effect estimate in favour of the interventions for the Body Mass Index (BMI) for multicomponent (BMI − 0.32 (95% CI: -0.54, -0.09) kg/m², BMI z-Score − 0.07 (95% CI: -0.14, -0.001)) and single-component interventions (BMI − 0.14 (95% CI: -0.21, -0-06) kg/m²; BMI z-Score − 0.05 (95% CI: -0.10, -0.01)) [14].

The WHO describes BMI as the most useful, albeit rough measure at the population level [8]. However, it does not distinguish between fat mass and fat free mass, nor between subcutaneous and visceral fat, nor between abdominal and general adiposity. Visceral fat in particular is highly metabolically active [15, 16] and is recognized as an important risk factor for many chronic diseases (including type 2 diabetes and cardiovascular disease), as well as symptom severity in COVID-19 [17, 18].

Simple methods to measure abdominal obesity are waist-circumference (WC), waist-to hip-ratio and waist-to height-ratio. Especially regarding children WC is recommended for global obesity surveillance and clinical practice [19, 20]. Compared to BMI, WC has a similar [21] or improved ability to predict health outcomes in adults [16, 19, 22–26] as well as risk factors for cardiovascular disease in children [27]. WC is a precise and slightly earlier indicator for the development of metabolic syndrome in adulthood [28]. Rather than BMI, WC is associated with motor performance in kindergarden children [29]. It can be a useful tool in diagnosing metabolic syndrome in childhood and adolescence [30, 31], as well as for detecting early lifestyle modifications [32]. Nevertheless, only a few reviews in related areas of research have explicitly considered WC as an outcome parameter [33–35].

To the best of our knowledge, this is the first systematic review and meta-analysis to examine the effect of school-based obesity prevention interventions on abdominal obesity, operationalised as WC. In addition, our study aimed to provide new insights into the field, for example regarding the quality of studies.

## Materials and methods

This review is an update of a comprehensive systematic review on prevention of overweight and obesity in schools [36], restricted to the outcome parameter WC. Our search was conducted at the end of 2019. The former literature search, conducted in December 2015, had a broader perspective in terms of evaluating different anthropometric outcome parameters including BMI, body fat percentage, and WC. In the former review 48 primary studies were included and data qualitatively summarised. Most studies (*n* = 38) reported data on changes in mean BMI over time and predominantly showed effects in favour of the intervention up to -0.2 kg m².

Former and current literature searches as well as the reviewing process were performed according to the Cochrane Handbook of Systematic Reviews of Interventions [37] and the Preferred Reporting Items for Systematic Reviews and Meta-Analyses (PRISMA) statement [38].The current search was adapted from the previous one in terms of criteria and execution, in order to avoid distortions due to deviations. A study protocol was not registered or published.

### Search

Nine databases were searched comprehensively and systematically at the end of 2019: BIOSIS Preview, Cochrane Library, DARE & NHSEED, DAHTA, Embase, HTA (INAHTA), MEDLINE and PubMed [see Additional file 1]. The search strategy was aligned to the previous review and contained terms in relation to participants, school setting, prevention and health promotion, overweight and obesity, intervention and anthropometric outcomes [36]. Full search strategies for all data bases are presented in detail in additional tables [see Additional file 1]. The reference lists of the retrieved full texts were searched for additional relevant publications. Our final search included publications from the period 2015 to 2019.

### Eligibility criteria

Inclusion criteria were as follows: (1) controlled trials with or without randomisation, (2) school as the setting for intervention, (3) participants aged 6 to 18 years, (4) studies assessed students‘ WC in cm or standard deviation (SD) / z-score, (5) interventions aimed at the prevention of overweight and obesity, (6) comparison groups were active controls, usual practice controls or wait-list controls, and (7) an English or German version of the full-text publication was available. Exclusion criteria comprised: (1) interventions designed for treatment of overweight or obesity, (2) studies included only overweight or obese participants, and (3) studies targeted specified groups with certain (chronic) diseases such as diabetes.

### Study selection

Regarding the current literature search, studies were imported to Citavi 5. Duplicates were identified and one reviewer (AK) screened titles, abstracts and full texts according to inclusion and exclusion criteria. A second reviewer (KB) screened a random sample (titles 10%, abstracts 10%, full texts 20%) generated by an online random number generator [39]. Deviations were checked. Any discrepancies were resolved by discussion or consultation with a third reviewer. Studies from the previous review that reported data for WC were included.

### Data extraction

For each study data were extracted covering information on authors, publication year, country of origin, study design, sample characteristics, outcome assessment, intervention components and period as well as statistical analysis methods and relevant results. Data extraction was based exclusively on the data reported in the included publications and conducted by the first reviewer (AK) using Microsoft Excel and the Cochrane Public Health Group Data Extraction Template 0–1 [40].

### Study appraisal

The risk of bias was assessed for all included studies (AK) regarding the outcome difference in mean change in WC over time. According to study design, the Cochrane Risk of Bias Tool Version 2 (RoB 2) [41, 42] was used for individual-randomised controlled trials, the adapted RoB 2 test version for cluster-randomised controlled trials (RoB 2 Cluster) [43] and the Cochrane Risk of Bias in non-randomised studies of intervention Tool (ROBINS-I) for non-randomised controlled trials [44, 45]. When assessing various domains of potential for risk of bias, each study was judged with an overall score, rated as ‘low’, ‘high’ or of ‘some concern’ for RoB 2 and as ‘low’, ‘moderate’, ‘serious’ or ‘critical’ for ROBINS-I.

### Data synthesis

#### Overview of studies and evaluated interventions

A qualitative overview was structured by extracted data on country of origin, specifics of study design and analysed sample, as well as on intervention features. Studies were categorised by geographic region and by income level according to the World Bank classification of country of origin at study start [46]. Age groups were defined according to the populations included in the individual studies. Interventions were categorised by intervention period and intervention components.

### Meta-analyses

Quantitative summaries were performed by conducting meta-analyses using Review Manager (RevMan version 5.4 software) [47]. Randomised as well as non-randomised studies were included, if the study design allowed the research subject to be addressed [48]. Given the higher potential for bias in cluster-randomised and especially non-randomised studies, studies assessed as having high or serious potential for risk of bias were excluded [48, 49]. Separate meta-analyses were performed for the two different outcomes of WC measured in cm or as WC z-score.

According to Higgins et al. [49], results of cluster (randomised) trials were considered for a meta-analysis if a direct effect estimate was reported for the required effect measure, including standard error or confidence interval (CI). Other necessary conditions for inclusion were consideration of cluster design in the statistical analysis (usually based on a multilevel model or generalised estimated equation), adjustment for baseline value of the required effect measure, and reporting effects for the total sample [49]. Only reported aggregated data were taken into account.

Considering the change in WC as a continuous outcome, we aimed to use mean differences between the baseline and post-intervention values of intervention and control groups to indicate effect sizes. Assuming a diversity in studies and interventions, e.g., regarding sample characteristics and intervention specifics, outcomes were combined in the meta-analyses using the inverse variance random-effects model [50, 51]. Sensitivity analyses were conducted based on non-overlapping 95% CI, study specifics such as sample size or using a fixed effect analysis where indicated. For further exploration, subgroup analyses were conducted for possible moderating factors, such as study design, age groups or intervention characteristics like intervention type and period [51]. In order to estimate publication bias funnel plots were applied if ten or more studies were included in a meta-analysis [52].

## Results

### Screening results

**Fig. 1:**
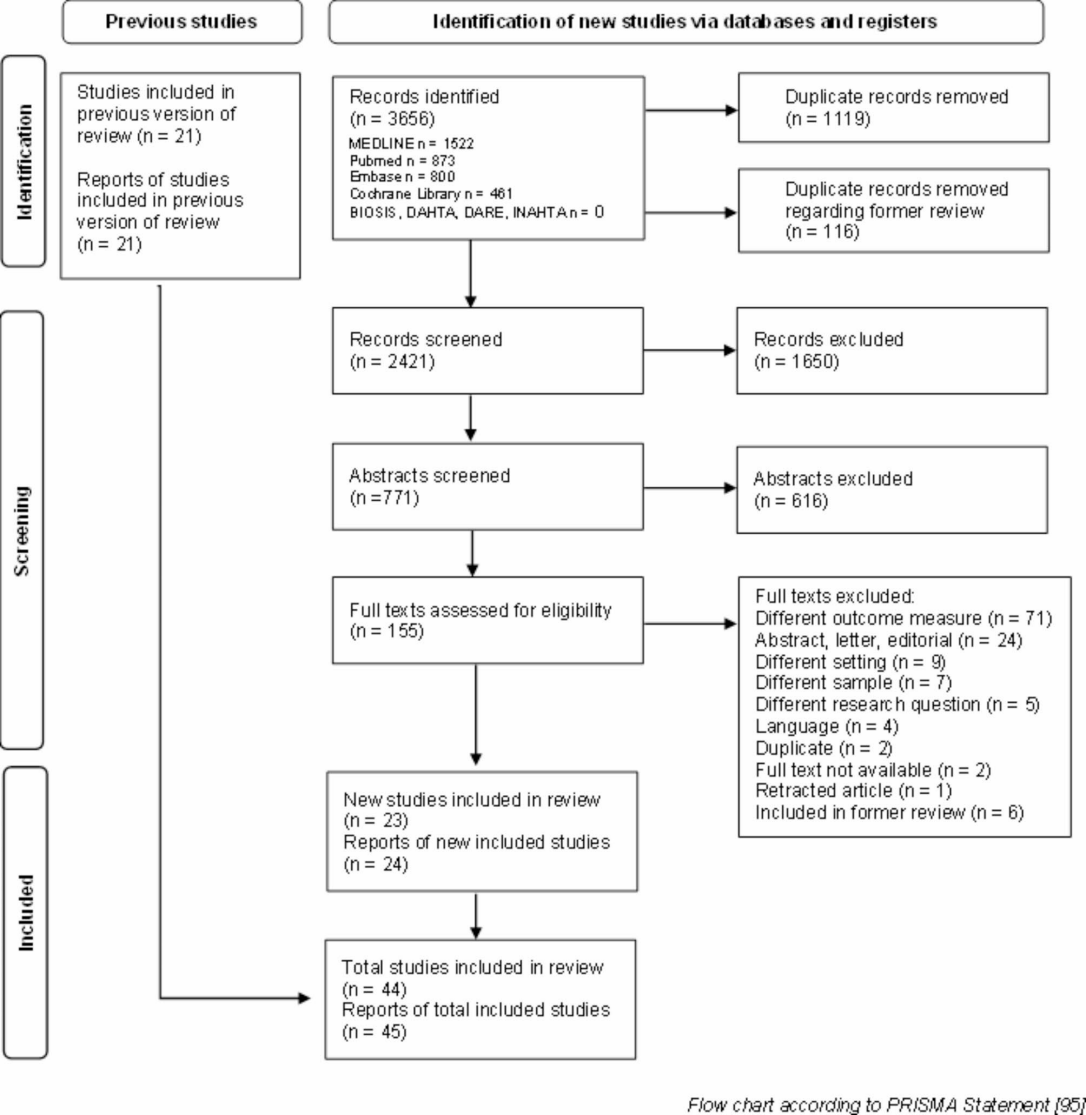
PRISMA Flow chart of screening process

In the current search, we identified 3656 relevant records of which 1119 were excluded as duplicates. A comparison with the results of the previous review revealed further 116 records as doublets. 2421 titles, 771 abstracts and 155 full texts were screened. Finally, 24 publications [53–76] on 23 studies from the current search, as well as 21 studies [77–97] from the former review [36] fulfilled the inclusion criteria, comprising a total of 39.837 participants. The flow chart of the screening process is presented in Fig. 1 according to the PRISMA Statement [98]. A comparison of the screening results for the random samples showed an interrater reliability of 88.3% (screening of titles), 92.2% (screening of abstracts) and 96.8% (screening of full texts). Deviations were explored and eventually resulted in a more sensitive screening strategy for the first reviewer (AK).

### Data extraction

Details for each individual study regarding study and design characteristics, key components of intervention and considered study results are presented in supplementary material for randomised controlled trials [see Additional file 2] and for non-randomised controlled trials [see Additional file 3].

### Study appraisal

Risk of bias was assessed with a suitable tool according to study design as described in the methods section. We identified one individual–randomised controlled trial [70], 29 cluster-randomised controlled trials [53, 54, 56, 58, 59, 61, 63–67, 71, 75, 78, 80–83, 85, 87–91, 93–97] and 14 non-randomised controlled trials [55, 57, 60, 68, 69, 72–74, 76, 77, 79, 84, 86, 92].

### Study appraisal: randomised controlled trials

**Fig. 2:**
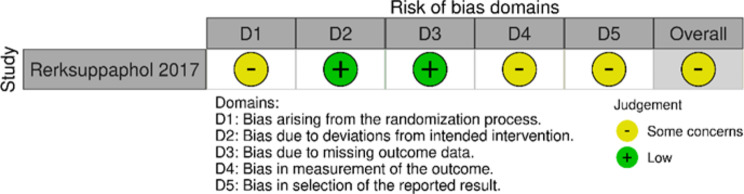
RoB 2 for individual-randomised controlled trials

**Fig. 3:**
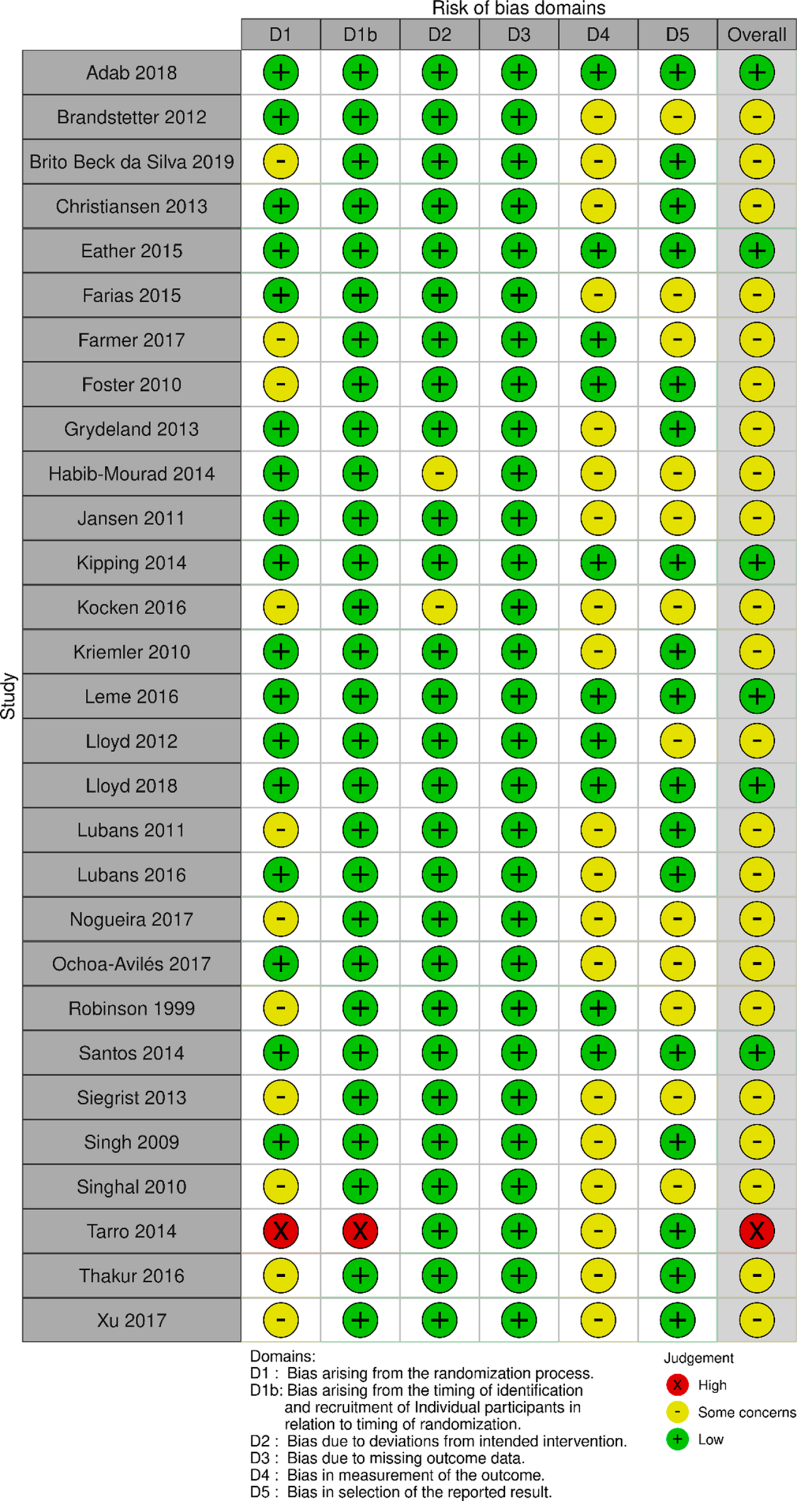
RoB 2 for cluster-randomised controlled trials

Assessment of risk of bias for each study using randomisation procedures is presented in Figs. 2 and 3 using robvis [99]. Only six studies [53, 56, 63, 64, 87, 93] were rated as having a low potential for risk of bias, all of which were cluster-randomised controlled trials. Most of the studies using a randomised design [54, 58, 59, 61, 65–67, 70, 71, 75, 78, 80–83, 85, 88–91, 94–96] (*n* = 23) were classified as of ‘some concern‘ in terms of risk of bias; mostly due to possible bias in measurement of outcomes (n = 20), given non-blinding of assessors (n = 8) or no information presented on this subject (n = 12), possible bias of selection in the reported results (n = 13) due to no information on a pre-specified analysis plan, and possible bias arising from the randomisation process (n = 12) mainly because of insufficient description (n = 11). One of these studies [97] was rated as having a high risk of bias, mainly due to the recruitment process.

### Study appraisal: non-randomised controlled trials

**Fig. 4:**
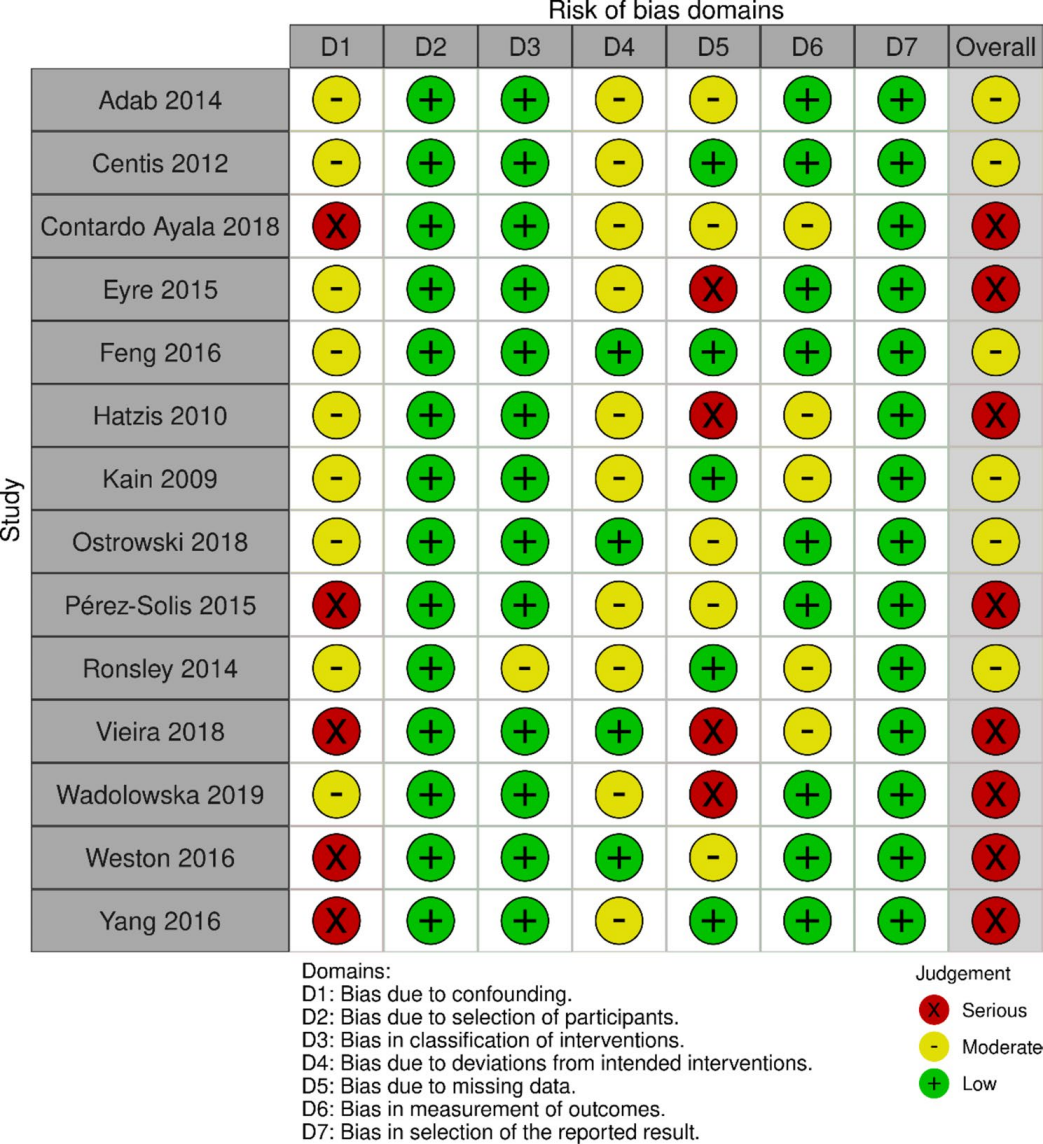
Risk of bias non-randomised controlled trials – ROBINS-I

**Fig. 5:**
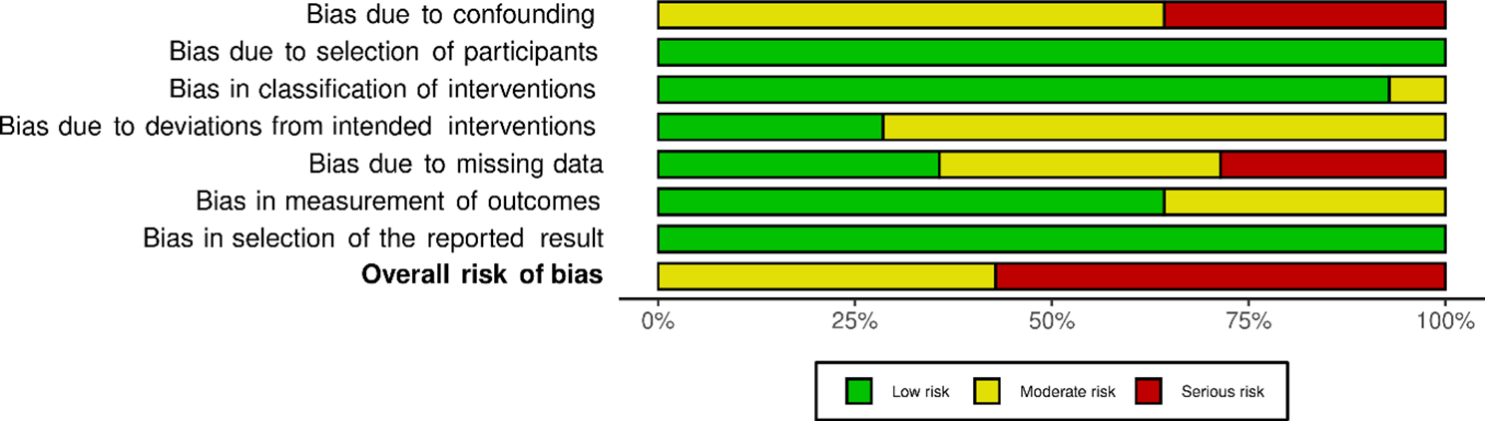
Robvis_Summary unweighted barplot ROBINS-I

Assessment of risk of bias for each individual non-randomised study is presented in Fig. 4 using robvis [99]. None of these studies was rated as having a low potential for risk of bias. Not even half of them [60, 68, 77, 79, 86, 92] (*n* = 6) were assessed as showing a moderate potential for risk of bias, due to a possible moderate confounding bias in all of them, possible moderate bias because of deviations from intended interventions (*n* = 4), possible moderate bias in measurement of outcome (*n* = 2), and possible moderate bias due to missing data (*n* = 1). Most of the studies [55, 57, 69, 72–74, 76, 84] assessed with ROBINS-I (*n* = 8) were classified as having a serious potential for risk of bias, due to a serious risk of bias regarding confounding (*n* = 5), and/or due to a serious risk of bias due to missing data (*n* = 4). A summary of assessment of risk of bias of these studies is illustrated in Fig. 5.

### Data synthesis

#### Characteristics of included studies

An overview of the included studies regarding design, sample specifics and intervention characteristics is presented in Table 1, complemented by an overview differentiated by study design.

**Table 1:** Characteristics of included studies

Study design	All		Cluster-RCT			NRCT			IRCT		
	n	%*	n	%*	References	N	%*	References	n	%*	References
	44	100	29	65,9	[53, 54, 56, 58, 59, 61, 63–67, 71, 75, 78, 80–83, 78, 80–91, 93–97]	14	31,8	[55, 57, 60, 68, 69, 73, 74, 76, 77, 79, 84, 86, 92]	1	2,3	[70]
**Country of origin**											
Europe	21	47,7	13	44,8	[53, 61, 64, 78, 80, 82, 85, 87–89, 94, 95, 97]	8	57,1	[57, 69, 72–74, 77, 79, 84]	0		
North America	6	13,6	3	10,3	[81, 91, 93]	3	21,4	[60, 68, 92]	0		
Australia/New Zealand	6	13,6	5	17,2	[56, 59, 65, 66, 90]	1	7,1	[55]	0		
Other	11	25,0	8	27,6	[54, 58, 63, 67, 71, 75, 83, 96]	2	14,3	[76, 86]	1	2,3	[70]
Income level of country **											
High income	33	75,0	21	72,4	[53, 56, 59, 61, 64–66, 78, 80–82, 85, 87–91, 93–95, 97]	12	85,7	[55, 57, 60, 68, 69, 72–74, 76, 77, 79, 92]	0		
Upper Middle income	9	20,5	6	20,7	[54, 58, 63, 67, 75, 83]	2	14,3	[84, 86]	1	2,3	[70]
Lower Middle income	1	2,3	1	3,5	[71]	0	0,0		0		
Lower income	1	2,3	1	3,5	[96]	0	0,0		0		
**Age of participants (years)**											
Predominantly < 9	12	27,3	8	27,6	[53, 59, 75, 78, 91, 93, 94, 97]	4	28,6	[60, 69, 77, 84]	0		
Predominantly 9 to 13	19	43,2	12	41,4	[61, 64–67, 80–83, 87, 89, 95]	7	50,0	[57, 68, 72, 73, 76, 79, 86]	0		
Predominantly > 13	9	20,5	7	24,1	[54, 56, 58, 63, 71, 90, 96]	2	14,3	[55, 76]	0		
Other	4	9,1	2	6,9	[85, 88]	1	7,1	[92]	1	2,3	[70]
**Gender of participants**											
Just girls	1	2,3	1	3,5	[63]	0	0,0		0		
Just boys	2	4,6	2	6,9	[65, 90]	0	0,0		0		
**Outcome parameter**											
WC (cm)	35	79,6	22	75,9	[54, 56, 58, 59, 63, 65–67, 75, 78, 80–83, 85, 90, 91, 93–97]	12	85,7	[55, 60, 68, 69, 72, 74, 76, 77, 79, 84, 86, 92]	1	2,3	[70]
WC z-score	7	15,9	6	20,7	[53, 61, 64, 71, 87, 88]	1	7,1	[73]	0		
Both	2	4,6	1	3,5	[89]	1	7,1	[57]	0		
**Intervention period**											
< 1 academic year	14	31,8	8	27,6	[56, 63, 65, 71, 83, 90, 91, 96]	5	35,7	[55, 57, 68, 73, 74]	1	2,3	[70]
1 academic year	20	45,5	15	51,7	[53, 54, 58, 64, 66, 75, 78, 80, 85, 87–89, 93–95]	5	35,7	[72, 76, 77, 79, 92]	0		
> 1 academic year	10	22,7	6	20,7	[59, 61, 67, 81, 82, 97]	4	28,6	[60, 69, 84, 86]	0		
**Intervention type**											
PA + Diet	30	68,2	19	65,5	[53, 54, 61, 63, 64, 75, 78, 81–83, 85, 87, 89, 90, 93–97]	10	71,4	[57, 60, 68, 73, 76, 77, 79, 84, 86, 92]	1	2,3	[70]
PA + Diet + Screen time	3	6,8	1	3,5	[71]	2	14,3	[69, 72]	0		
Exclusively PA	8	18,2	7	24,1	[56, 58, 59, 65, 66, 80, 88]	1	7,1	[74]	0		
Exclusively Diet	1	2,3	1	3,5	[67]	0	0,0		0		
Sedentary time	1	2,3	0	0,0		1	7,1	[55]	0		
Screen time	1	2,3	1	0,0	[91]	0	0,0		0		
**Intervention: specific components**											
Teacher training	18	40,9	11	37,9	[53, 63, 65, 67, 71, 75, 78, 87, 91, 93, 94]	7	50,0	[55, 60, 69, 77, 84, 86, 92]	0		
Family involvement	15	34,1	11	37,9	[53, 61, 67, 71, 75, 83, 87, 89–91, 94]	4	28,6	[60, 69, 77, 79]	0		
Family information	13	29,5	9	31,0	[54, 63–65, 78, 81, 82, 85, 96]	4	28,6	[68, 76, 84, 86]	0		
Pupils with ow or obesity	3	6,8	1	3,5	[85]	2	14,3	[60, 76]	0		

IRCT = individual-randomised controlled trial, NRCT = non-randomised controlled trial; RCT = randomised controlled trial; ow = overweight; PA = physical activity; WC = waist circumference

* Differences in proportions due to rounded results

** Due to World Bank classification at start of intervention

Most of the included studies (*n* = 29; 65.9%) were cluster-randomised controlled trials. The largest proportion was implemented in high-income countries (*n* = 33; 75%), mainly in Europe (*n* = 21; 47.7%). Almost half of the studies focused on children and adolescents aged 9 to 13 years (*n* = 19; 43.2%), a quarter on children mainly younger than 9 years (*n* = 12; 27.3%) and slightly fewer on adolescents older than 13 years (*n* = 9; 20.5%). In four studies a different age range was reported. Nearly all studies included boys and girls (*n* = 41; 93.2%), two studies addressed exclusively boys, one study exclusively girls. Predominantly the studies reported WC measured in cm (*n* = 35; 79.6%), with only two studies reporting z-score as well [57, 64].

Regarding intervention characteristics, the largest proportion of the included studies (*n* = 20; 45.5%) reported an intervention period of one academic year, the smallest (*n* = 10; 22.7%) of a longer duration. Two thirds of the included studies (*n* = 30; 68.2%) combined intervention measures focusing on PA and diet. Eight studies (18.2%) focused exclusively on PA. Nearly half of the evaluated interventions (*n* = 18; 40.9%) explicitly comprised training for teachers or other school staff, a third (*n* = 15; 34.1%) included the additional involvement of pupils’ families, and more than one quarter (*n* = 13; 29.5%) offered information material for families. Very few interventions (*n* = 3; 6.8%) contained an additional component regarding children or adolescents with overweight or obesity.

Restricting the overview to the studies from the current search, slightly less than half of these studies [55, 57, 60, 68, 69, 72–74, 76] (*n* = 9 out of 23) had a non-randomised controlled design. Furthermore, one third [54, 58, 63, 67, 71, 75, 76] (*n* = 7) of these trials were performed within countries in Middle and South America or in Asia.

Although nearly all studies used a cluster design, especially the randomised trials, only two studies reported an intra-cluster correlation coefficient (ICC) [80, 88] which compares the within-group variance with the between-group variance [51, 100].

Regarding WC as outcome parameter overall, two thirds of the included studies (*n* = 30) reported effects in favour of the experimental group [54–59, 61, 63, 64, 66, 67, 70–75, 77, 78, 81, 85, 87–89, 91, 93–97], 12 studies found no statistical significant effects [53, 60, 65, 68, 69, 79, 82–84, 86, 90, 92], and two studies reported effects in favour of the control group [76, 80].

### Meta-analyses

#### Outcome parameter WC in cm

13 studies [56, 59, 63, 65, 67, 75, 77, 78, 80, 89–91, 94] fulfilled the criteria for inclusion in a meta-analysis regarding WC measured in cm. Cluster-randomised controlled trials made up the largest share [56, 59, 63, 65, 67, 75, 78, 80, 89–91, 94] (*n* = 12). Of these, two studies [56, 63] were categorised as having low potential for risk of bias, all others as of ‘some concern’. One study [77] did not use a randomised design, and was rated as having a moderate potential for risk of bias. In all but one study [56] ‘school’ was the cluster variable and, if applicable, the unit of randomisation. Most of the studies [56, 59, 65, 77, 78, 80, 89–91, 94] (*n* = 10) were performed in high-income countries, three [63, 67, 75] in upper middle income countries. One study [63] exclusively addressed girls, two studies [65, 90] exclusively boys. The biggest proportion [59, 75, 77, 78, 91, 94] (*n* = 6) included pupils predominantly younger than nine years, four studies [65, 67, 80, 89] included pupils aged nine to thirteen years, and three studies [56, 63, 90] included predominantly older students. Six of the evaluated interventions [75, 77, 78, 80, 89, 94] lasted about one academic year, five [56, 63, 65, 90, 91] were of a shorter period, and two [59, 67] of a longer one. Combined features on the subjects of PA and diet are reported in seven of these trials [63, 75, 77, 78, 89, 90, 94], with the other six evaluating interventions focused on a single topic: PA [56, 59, 65, 80], diet [67] or screen time [91]. Additional information for the families was reported in ten studies [63, 65, 67, 75, 77, 78, 89–91, 94], seven of them [67, 75, 77, 89–91, 94] also involved family members in individual intervention measures. Active teacher training was part of eight intervention programs [63, 65, 67, 75, 77, 78, 91, 94].

**Fig. 6:**
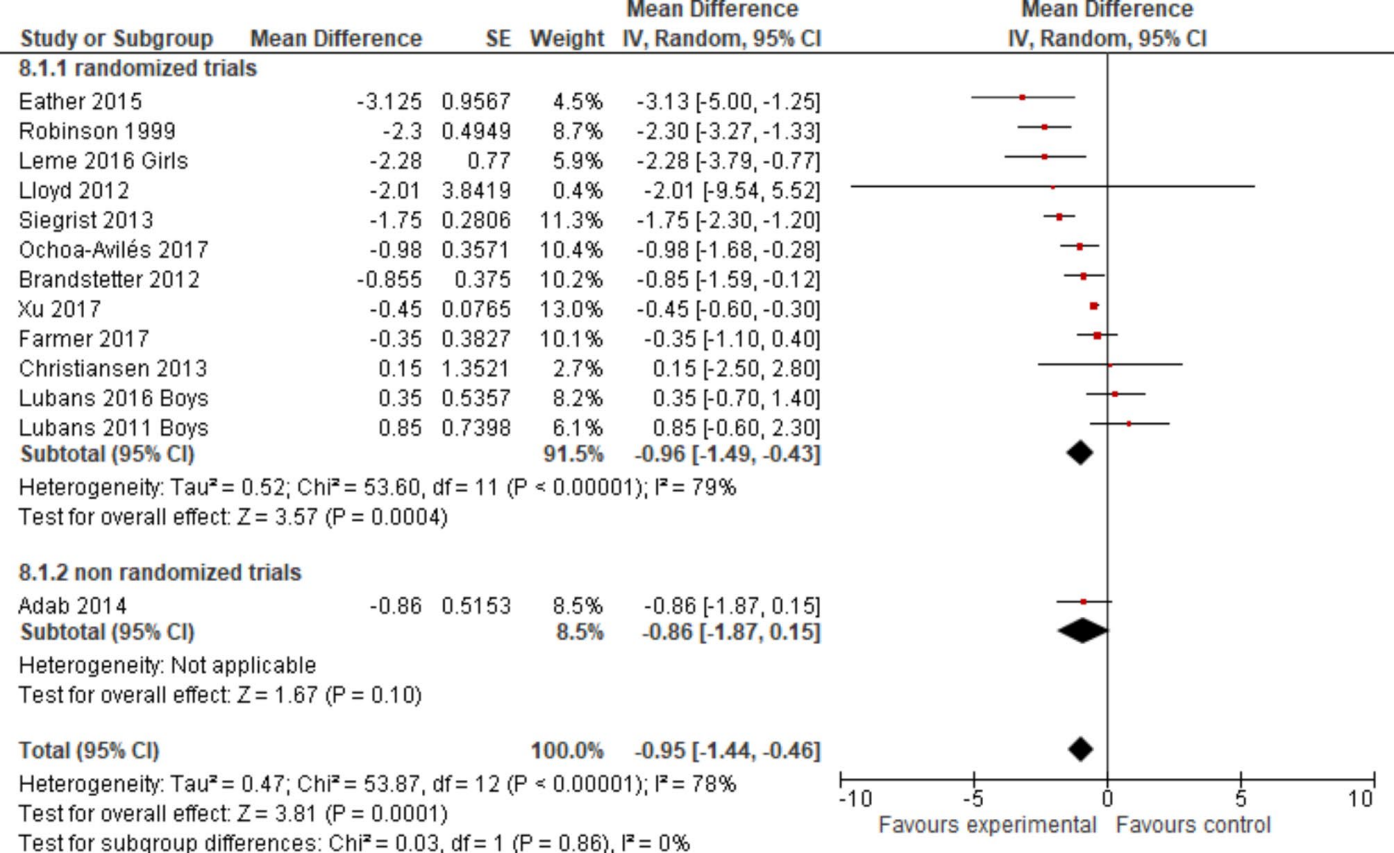
Forest plot displaying the mean differences of school-based interventions on WC measured in cm - sorted by effect size

The standard error as a statistical measure was presented for one of these trials [63]; for all others the reported data on the 95% CI provided the basis to calculate the meta-analysis (Fig. 6).

The overall pooled effect for the difference in mean change of WC in cm (95% CI) was -0.95 (-1.87; -0.46) in favour of the experimental group, reaching the level of statistical significance with a p-value < 0.05 (Fig. 6). The p-value of the Chi² test with *p* < 0,0001 confirmed the presence of statistical heterogeneity. I² was 78%, suggesting 78% of the variability in treatment effect was due to real study differences and only 22% due to chance. Visual inspection showed a relatively wide scatter of effect estimates with little overlap of confidence intervals, which lead to the same interpretation. The variance in the true outcomes across studies is estimated by Tau². In this analysis we found a Tau² of 0.47, indicating heteroegeneity which can be evaluated as moderate. The forest plot in Fig. 6 already included subgroup differentiation according to randomised or non-randomised study design. For the subtotal of cluster-randomised controlled trials, the effect estimate was just very slightly different: WC (cm) (95% CI) -0.96 (-1.49; -0.43) with a p-value < 0.05, indicators of statistical heterogeneity were quite similar to the overall results.

**Fig. 7:**
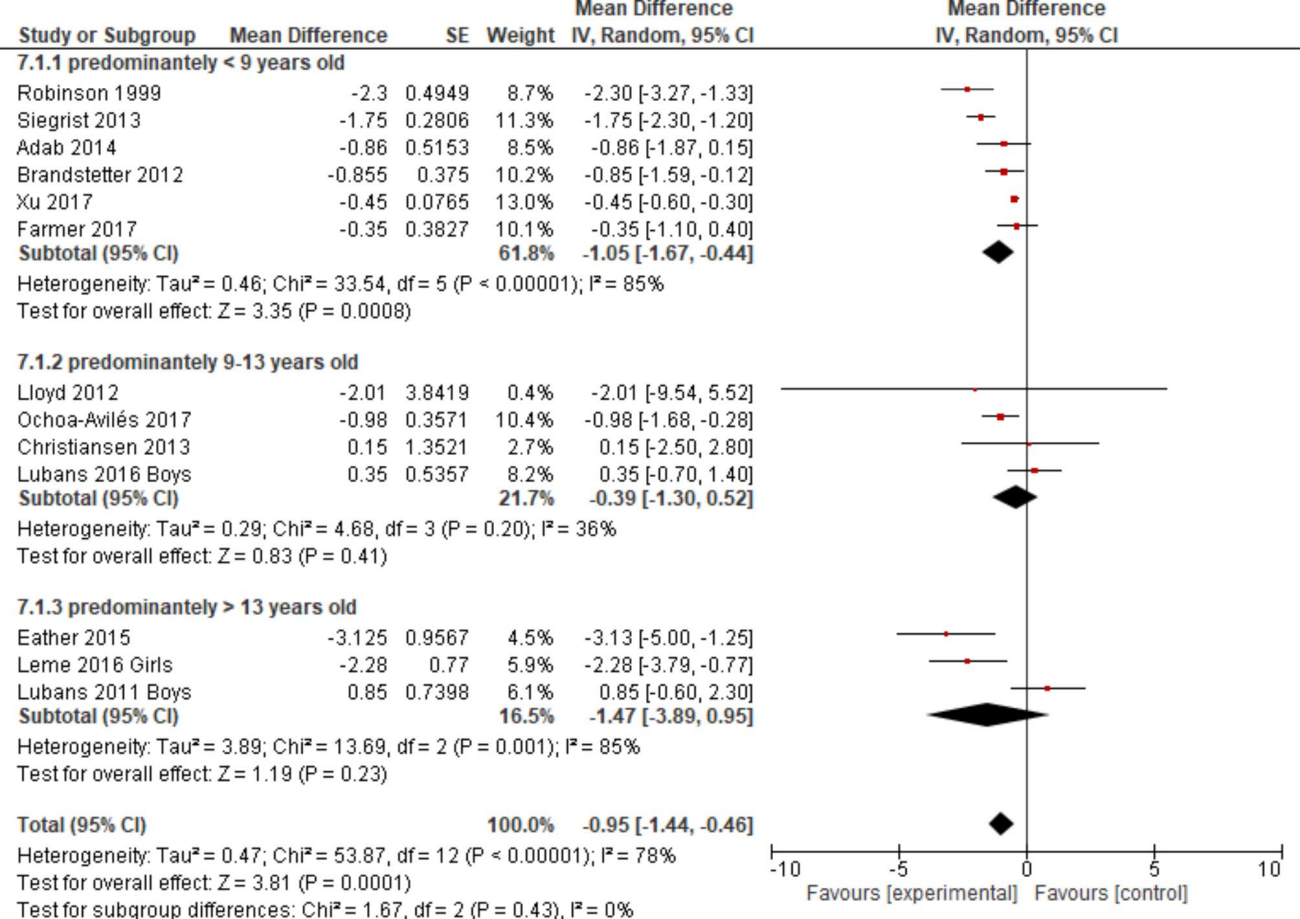
Forest plot (WC n cm) regarding age groups sorted by effect size

**Fig. 8:**
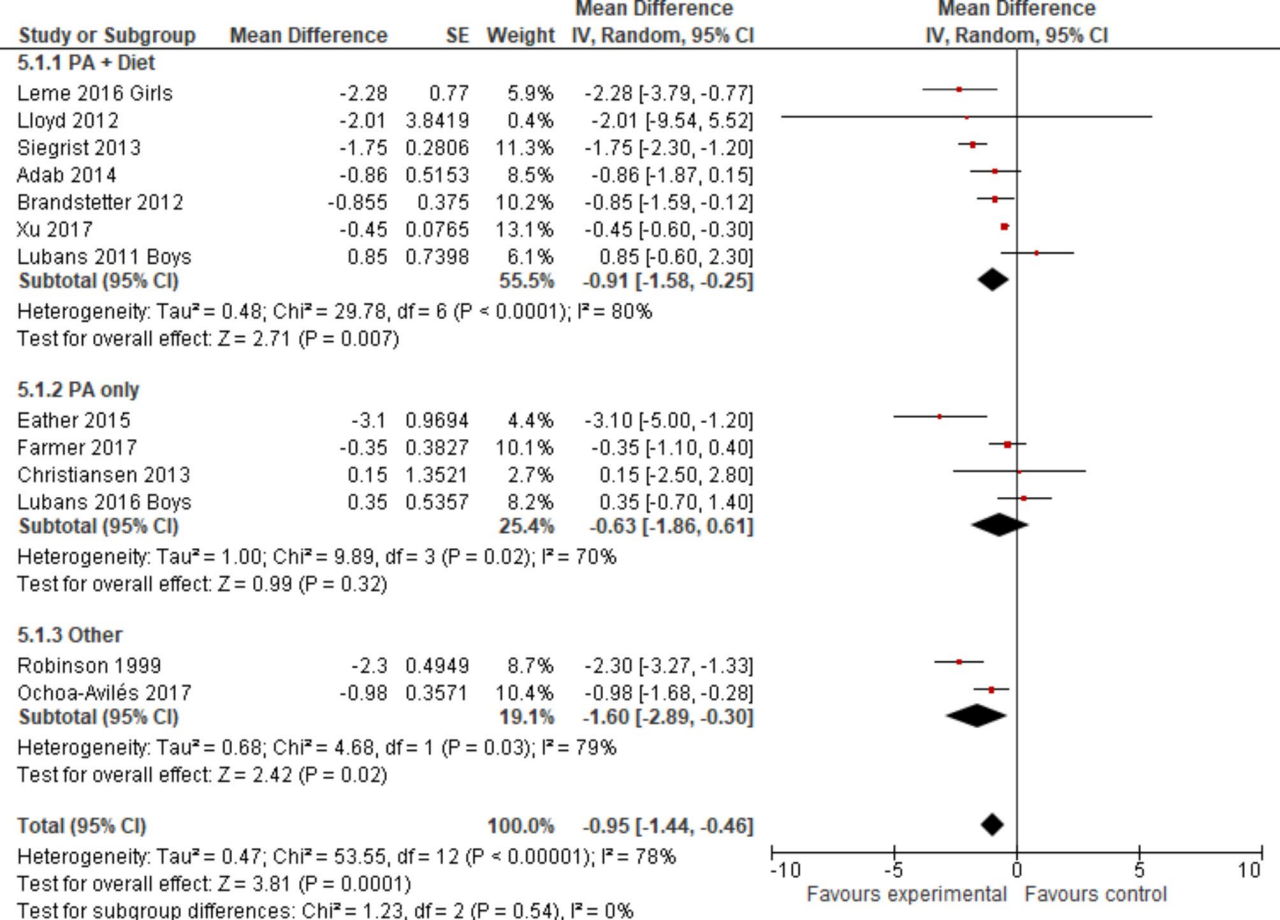
Forest plot (WC in cm) intervention type sorted by effect size

**Fig. 9:**
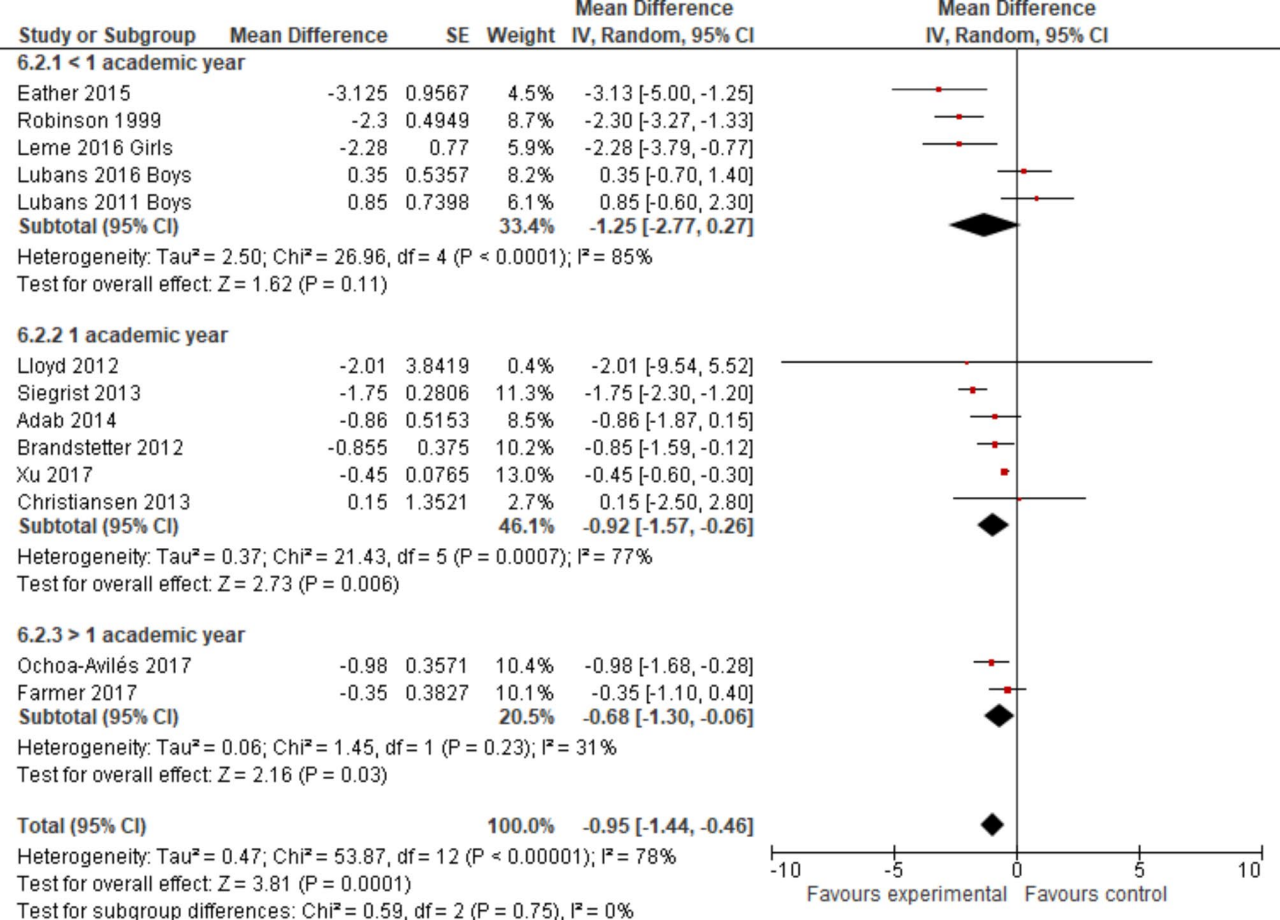
Forest plot (WC in cm) intervention period sorted by effect size

Heterogeneity was further explored in subgroup analyses regarding age group (Fig. 7), intervention type (Fig. 8) and intervention component (Fig. 9). None except one of the subgroup analyses showed considerably different results in terms of statistical measures, if more than two studies were included. For the subgroup of children aged nine to thirteen years, a p-value for Chi²-test of *p* = 0.20 indicated no presence of statistical heterogeneity, an I² of 36% represented a lower proportion of total variability due to between-study heterogeneity and a slightly lower Tau² of 0.29 was reached. Confidence intervals did overlap considerably.This subgroup analysis resulted in a small overall effect in favour of the experimental group of WC z-score mean -0.39 (-1.30; 0.52) and a p-value > 0.05. The biggest pooled effect size was reached for studies aimed at students older than 13 years: WC (cm) (95% CI) -1.47 (-3.89; 0.95) (Fig. 7), which did not reach the level of statistical significance with a wide 95% CI. Regarding sensitivity analyses, the change of pooled effects was not relevant in terms of size.

**Fig. 10:**
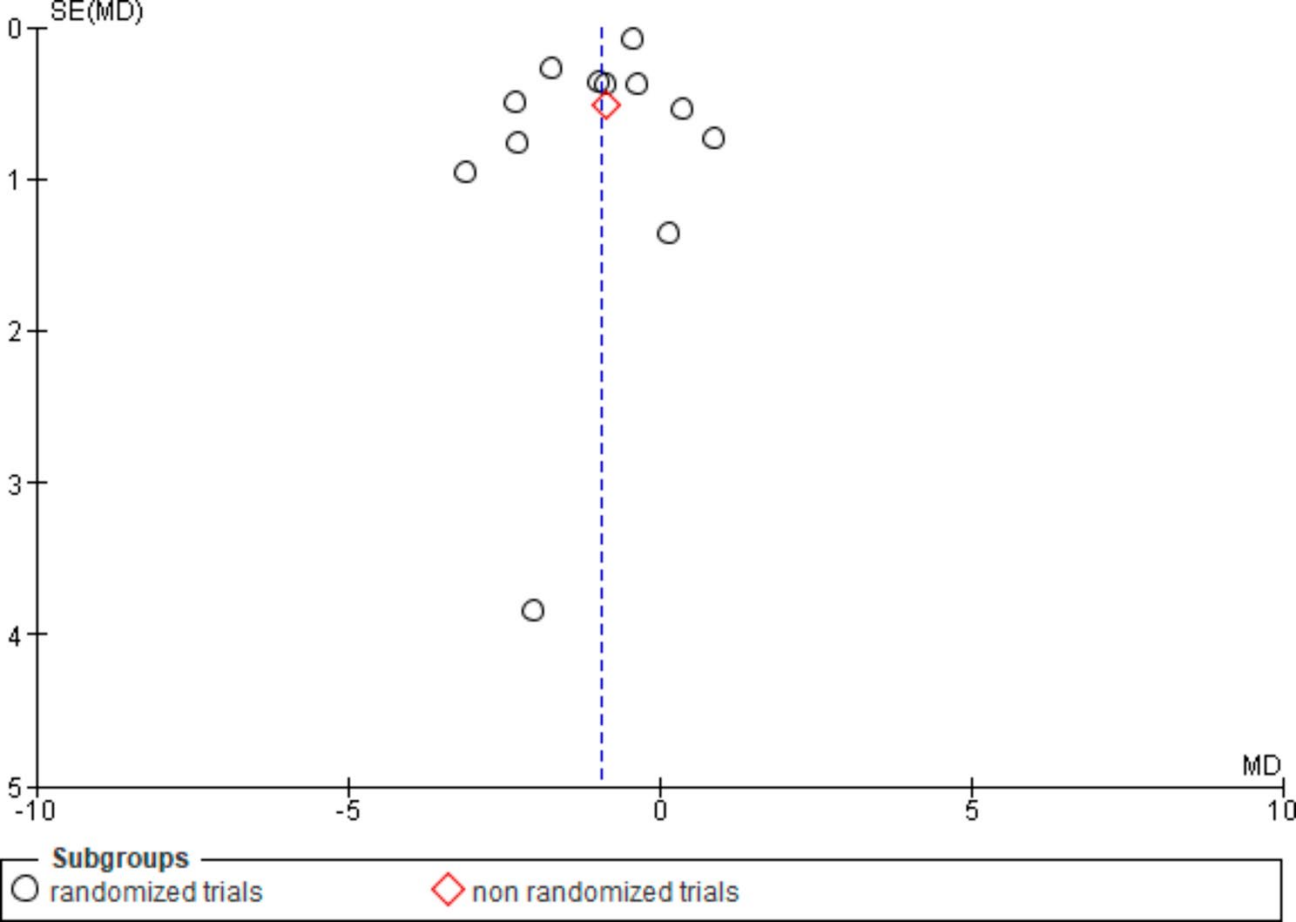
Funnel plot for included study results based on WC measured in cm

A funnel plot was created for the meta-analysis regarding results for WC measured in cm (Fig. 10) since more than ten studies were included. Visual examination did not indicate a strong asymmetrical distribution of included results.

### Outcome parameter: WC z-score

**Fig. 11:**
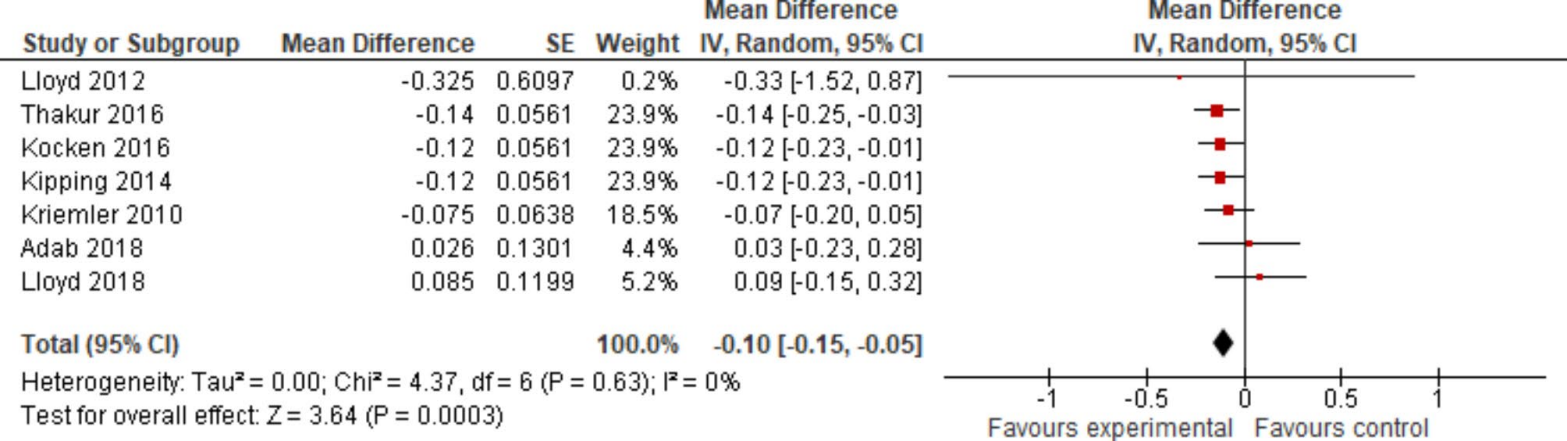
Forest plot displaying the mean differences of school-based interventions on WC z-score - sorted by effect size

Seven studies [53, 61, 64, 71, 87–89] reported overall effect estimates and 95% CI for WC z-score, which could be pooled in a separate meta-analysis, presented in Fig. 8. All of them were cluster-randomised controlled trials, with school as the unit of randomisation. Of these, three studies [53, 64, 87] were categorised as having low potential for risk of bias, all others as of ‘some concern’. Six of these studies were performed in high income European countries [53, 61, 64, 87–89]. All focused on boys and girls, most of them [61, 64, 87, 89] (*n* = 4) aged nine-to-thirteen years old. Intervention features combined PA and diet in five of these studies [53, 61, 64, 87, 89] and lasted about one academic year in another five studies [53, 64, 87–89]. The overall pooled effect, as reported in Fig. 11, favoured the experimental group with an effect size for difference of mean change in WC z-score (95% CI) -0.10 (-0.15; -0.05). The effect reached the level of statistical significance but should still be categorised as small. The p-value of the Chi² test with *p* = 0.63, an I² of 0% and a Tau² = 0.00 suggested that statistical heterogeneity was not present or at least could be evaluated as low. A narrow scatter of effect estimates and considerably overlapping confidence intervals, confirmed this result. Due to the inclusion of less than ten studies, a funnel plot was not created.

### Certainty of the body of evidence

On the assumption of observed estimates of intervention effects would differ from study to study due to existing clinical and methodological variability as well as sampling variety, we chose a random effects model for both meta-analyses. By far the biggest share (outcome parameter WC measured in cm) or all (outcome parameter WC z-score) of the included studies were randomised controlled trials. Risk of bias was rigorously assessed. Studies with a high or serious risk of bias were excluded. In the analysis for the outcome parameter WC measured in cm we rated statistical heterogeneity as present but moderate, in the analysis for the outcome parameter WC z-score as low. A funnel plot was created for the analysis of the outcome parameter WC measured in cm not indicating a relevant publication bias. Within our comprehensive literature search and within the analyses we identified and included studies reporting results in favour of the experimental and the control group or not reaching the level of statistical significance, which might indicate a low publication bias. Overall, we rated the results as robust, with slightly more limitations for the pooled effect estimate for the outcome parameter WC measured in cm, due to higher statistical heterogeneity. As for both pooled effect estimates the confidence interval did not contain zero, there was reliable evidence, that on average the intervention effects were beneficial.

## Discussion

According to the WHO 37 million children under the age of five and 390 million children and adolescents were overweight in 2022 [4]. The so-called obesity pandemic is recognised as a major global health threat. Being overweight is one of the most important risk factors for many non-communicable diseases (NCDs), including cardiovascular conditions, diabetes, cancers, neurological disorders, chronic respiratory illnesses, and digestive troubles. The Global Burden of Disease Risk Collaborators estimate that five million people died from NCDs in 2019 as a result of being overweight [5].

Childhood obesity is associated with a greater risk of developing NCDs or being obese in adulthood. At the same time, it affects school performance and quality of life. Stigma, discrimination and bullying have adverse psychosocial effects [8, 10, 101]. The worldwide Covid-19 pandemic had a negative impact on the prevalence of overweight and its risk factors, especially regarding children [102, 103]. Circumstances surrounding the pandemic, both in terms of containment measures and the incidence of disease, led to relevant changes in children’s living conditions [104].

However, the majority of overweight in children and adolescents can be prevented [2, 4]. Concerted efforts are needed to prevent overweight and obesity, especially in early life [105–107]. Strengthening schools should be part of this. It is therefore crucial to identify effective interventions, particularly for abdominal obesity.

In regard to health outcomes, it is of interest to distinguish between subcutaneous and visceral fat, as the latter is highly metabolically active [15, 16], or between abdominal and general adiposity. WC is a simple method to measure abdominal obesity and is recommended for global obesity surveillance and clinical practice, especially regarding children [19, 20]. The WHO Childhood Obesity Surveillance Initiative favours WC as a simple indicator [20]. With this in mind, WC as an endpoint should be of greater importance in future efficacy studies. Nevertheless, there are still challenges for which solutions need to be found. The measurement procedure of WC should be standardised more strictly [108, 109]. In addition, a common consensus on WC cutoff points for overweight still has to be defined [20]. Yet, WC has the potential to be a more accurate indicator for certain risk factors and chronic diseases [110].

This systematic review provides an overview of the research on school-based interventions to prevent obesity. We focused on WC as an outcome measure, understanding this parameter as a specific indicator of abdominal overweight. To our knowledge, WC has rarely been the focus of analysis of the effects of corresponding interventions.

44 studies met the inclusion criteria, most of which used a cluster-randomised design. The result of the risk of bias assessment indicated a certain robustness of the data for the included cluster-randomised trials. Regarding the non-randomised studies, the result differed considerably. The lack of a randomisation process alone lead to a possible bias potential with regard to confounding. Nevertheless, randomisation is not always possible in preventive trials. Still, corresponding studies contribute to the evidence base in prevention and health promotion.

Only one third of the identified studies could be included in the meta-analysis in terms of WC measured in cm, and even fewer for WC expressed as a z-score value according to the a priori set criteria. We assumed the existence of differences between the included studies, particularly with regard to the interventions and their implementation, and therefore used a random effects model for the meta-analyses. For the parameter WC measured in cm, statistical heterogeneity was indicated, but could be classified as moderate. The overall effect did reach statistical significance and was categorised as small. The meta-analysis pooling the effect estimates for WC z-score indicated statistical heterogeneity that could be evaluated as at least low. WC z-score is already adjusted for gender and age, which may account for the reduction in heterogeneity and increased comparability of trial results. However, although the overall effect size reached the level of statistical significance, it was still considered small.

A funnel plot was created for just one of the meta-analyses due to the recommendation that it should be comprised of at least ten studies. Strong asymmetry was not indicated visually, which can be interpreted as a low potential for publication bias. Considering the result of the overall review, publication bias could not be excluded in principle. However, the fact that the included studies report small effects or sometimes effects in favour of the control group suggested that the degree of publication bias tended to be low.

Most of the results were in favour of the experimental group, suggesting that school-based interventions to reduce childhood overweight and obesity are effective in principle. Predominantly, the reported effect sizes were categorised as small. Various related reviews support this result [14, 111–113]. It is debatable what effect sizes are achievable in the area of universal prevention or health promotion interventions [114, 115]. Grydeland et al. [82] explicitly refer to the possibility of a limited potential for effects due to the inclusion of predominantly ‘healthy’ persons - in this case children without overweight. Intended effects in a stronger form should only be expected after a long period of time.

Since we conducted our database search at the end of 2019, more recent publications were not included in the synthesis. To get an impression of the current study situation and to critically evaluate our results accordingly, we performed a search on PubMed on 28 July 2024 for the latest articles published since 2023, using the original search terms (AK, RB). Of the 114 titles identified, 22 publications dealt with relevant interventions, including two reviews of reviews [116, 117], five systematic reviews [118–122], and 15 individual studies [123–137]. The majority reported effects in favour of the experimental group on anthropometric outcomes, such as BMI, BMI z-score, or weight status. For the outcome parameter WC, results were reported in four of the individual studies, with effects in favour of the experimental group in three studies [125, 129, 136] and no significant effect in one study [130]. These results confirm, or at least do not contradict our analysis. Another update-study can nevertheless come to different results.

Preventive interventions to reduce the prevalence of overweight and obesity are often evaluated in studies using a cluster design [36, 113]. In particular, cluster-randomised controlled trials have become common for evaluating public health interventions since the turn of the century [138]. Reducing contamination bias and improving the ability to conduct large-scale trials are considered to be some of their greatest potentials [51, 139–141]. Recommendations for appropriate assessment of risk of bias and statistical evaluations have been updated [43, 49]. Nevertheless, the (meta-) analysis of cluster trials, randomised or not, poses particular methodological challenges, for example when pooling the results for continuous outcomes [49, 51, 139–142]. Therefore it is essential that certain statistical values, such as the ICC or the standard error, are reported as well as details of statistical analysis methods and adjustment procedures. The possibility of providing further information in supplementary documents offers an opportunity here. Regarding the included cluster-randomised controlled trials, the reporting is predominantly good but there is room for improvement.

Systematic reviews and meta-analyses strengthen the evidence base for prevention and health promotion. This could support policy decisions to promote preventive policies and activities. However, the usefulness and interpretability of the results of meta-analyses for decision-makers and practitioners is worth a discussion [143]. Given the complexity of cluster design studies, it seems reasonable to develop a ‘reliable translation’ of study results. Study registries such as the European Xchange prevention registry [144] or the U.S. registry Blueprints for Healthy Youth Development [145] can be cited as examples. Additional ways of presenting meta-study results that meet the needs of decision and policy makers should be discussed [107, 146].

Since overweight is most often caused by an imbalance in the individual´s energy balance, prevention is primarily aimed at developing a healthy lifestyle. The WHO characterises the prevention of obesity as a societal rather than an individual responsibility [4]. Eating habits and physical activity patterns are very much a result of environmental and social factors. Many of the latter are well known as risk factors for childhood overweight and obesity [147–151]. Schools themselves interact with other subsystems [152] and are part of an obesogenic environment, which is characterised by its complex structures [153–155]. Nevertheless, schools are in a unique position to address prevention and health promotion, since they can reach almost all children and, to some extent also their families. Waterlander et al. used causal loop diagrams to analyse obesity-related behaviours in adolescents [152]. As a very tangible result, the authors showed that schools have the potential to provide a balancing feedback loop in relation to unhealthy eating. Schools should be strengthened politically and structurally to improve their effectiveness in preventing childhood overweight and obesity. Our review has shown that schools are a setting for successful prevention of childhood overweight and obesity, including abdominal obesity.

### Limitations

Despite our best efforts, the literature search may have missed studies due to the search being restricted to the period until the end of 2019 as well as other limiting inclusion criteria like language of publication. Only a fraction of the included publications reported effects in a way that they could be considered in the meta-analyses. Due to limited resources, the review process and study appraisal was mainly done by one reviewer (AK), an independent second screening at all levels was carried out using a random sample.

#### Strengths

These limitations are mitigated by several strengths. The systematic review followed standardised guidance like the PRISMA statement and the recommendations from the Cochrane group. Risk of bias was rigorously assessed with established tools according to the study design. Here the Rob 2 tool for cluster-randomised controlled trials is to be particularly emphasised. The use of multiple, complementary databases and inclusion of studies over several decades allow for confidence in the conclusions.

## Conclusions

We conducted a systematic review and meta-analyses to explore the effects of schoolbased interventions aiming to prevent childhood and juvenile overweight and obesity, with regard to abdominal obesity operationalised by the outcomeparater WC. 44 studies were identified for inclusion. 13 studies fulfilled the criteria for inclusion in a meta-analysis regarding WC measured in cm with an overall pooled effect for the difference in mean change of WC in cm (95% CI) of -0.95 (-1.87; -0.46) in favour of the experimental group, reaching the level of statistical significance with a p-value < 0.05. Seven studies fulfilled the criteria for inclusion in a meta-analysis regarding WC reported as z-score with an overall pooled effect for the difference of mean change in WC z-score (95% CI) of -0.10 (-0.15; -0.05) in favour of the experimental group, reaching the level of statistical significance with a p-value < 0.05. As none of the confidence intervals contained zero, there is strong evidence, that on average the intervention effects were beneficial.

Understanding schools as a subsystem in a nowadays obesogenic environment, they provide a unique setting for comprehensive intervention measures aiming at the prevention of overweight and obesity. They can only develop their full potential if they are accompanied by further measures in politics and society. If measures to prevent obesity are shifted more strongly into the area of early childhood, including pregnancy and family planning, schools will have an important role to play in maintaining healthy lifestyles.

## Electronic supplementary material

Below is the link to the electronic supplementary material.

**Supplementary Material 1: Additional file 1.** Data bases and search strategies.

**Supplementary Material 2: Additional file 2.** Design and characteristics – randomised controlled trials.

**Supplementary Material 3: Additional file 3.** Design and characteristics – non-randomised controlled trials: included studies sorted alphabetically.

## Data Availability

The datasets used and analysed during the current study are available from the corresponding author on reasonable request.

## Abbreviations

BMI: Body Mass Index
CI: Confidence Interval
NRCT: Non randomised controlled trial
ICC: Intra-Cluster Correlation Coefficient
PA: Physical activity
PRISMA: Preferred Reporting Items for Systematic Reviews and Meta-Analyses
RCT: Randomised controlled trial
RoB: Risk of bias
ROBINS-I: Risk of bias in non-randomised studies of intervention
SD: Standard deviation
WC: Waist circumference
WHO: World Health Organisation
