# Supplementary material for: Waist circumference as a parameter in school-based interventions to prevent overweight and obesity - a systematic review and meta-analysis
Source: BMC Public Health. 2024 Oct 17;24:2864. doi: 10.1186/s12889-024-20354-7 (PMC11488270; doi:10.1186/s12889-024-20354-7)
Supplement: Supplementary file 1 — Supplementary Material 1: Additional file 1. Data bases and search strategies. [file 12889_2024_20354_MOESM1_ESM.pdf]

Additional file 1: Data bases and search strategies

Table S1: Searched data-bases

Data base	Date of search	Hits
BIOSIS Previews	20.11.2019	0
Cochrane Library	20.12.2019	461
DAHTA	21.11.2019	0
DARE & NHSEED	22.11.2019	0
Embase	25.11.2019	800
HTA (INAHTA)	25.11.2019	0
MEDLINE	26.11.2019	1522
Pubmed	25.11.2019	873

Table S2: Full search strategy PubMed

Nr.	Search Term	Hits
#1	Search ""Child""[Mesh]	1.862.530
#2	Search ""Adolescent""[Mesh]	1.972.202
#3	Search ""Students""[Mesh]	122.358
#4	Search (((((((kids*[Title/Abstract]) OR child*[Title/Abstract]) OR boy*[Title/Abstract]) OR girl*[Title/Abstract]) OR student*[Title/Abstract]) OR youth*[Title/Abstract]) OR teen*[Title/Abstract]) OR adolescen*[Title/Abstract]) OR adoleszen*[Title/Abstract]	1.896.377
#5	Search ((((((((Jung*[Title/Abstract]) OR Schüler*[Title/Abstract]) OR Schueler*[Title/Abstract]) OR Schuler*[Title/Abstract]) OR Kind*[Title/Abstract]) OR Mädchen[Title/Abstract]) OR Maedchen[Title/Abstract]) OR Jugend[Title/Abstract]) OR Jugendalter[Title/Abstract]) OR Jugendliche*[Title/Abstract]	174.464
#6	Search #1 OR #2 OR #3 OR #4 OR #5	3.790.819
#7	Search ""Schools""[Mesh]	114.244
#8	Search ((((((((((primary school*[Title/Abstract]) OR secondary school*[Title/Abstract]) OR high school*[Title/Abstract]) OR vocational school*[Title/Abstract]) OR schoolbased[Title/Abstract]) OR school based[Title/Abstract]) OR elementary school*[Title/Abstract]) OR grammar school*[Title/Abstract]) OR middle school*[Title/Abstract]) OR comprehensive school*[Title/Abstract]) OR public school*[Title/Abstract]) OR independent school*[Title/Abstract]	77.190
#9	Search (((((((((Grundschul*[Title/Abstract]) OR Realschul*[Title/Abstract]) OR Hauptschul*[Title/Abstract]) OR Schule*[Title/Abstract]) OR schulische[Title/Abstract]) OR Gymnasi*[Title/Abstract]) OR Primarstufe*[Title/Abstract]) OR Sekundarstufe*[Title/Abstract]) OR schulbasiert*[Title/Abstract]) OR Gesamtschul*[Title/Abstract]) OR Oberschul*[Title/Abstract]	653
#10	Search #7 OR #8 OR #9	176.646
#11	Search ""Obesity""[Mesh]	203.746
#12	Search ""Adiposity""[Mesh]	11.474
#13	Search (((((adiposity[Title/Abstract]) OR adipositas[Title/Abstract]) OR overweight[Title/Abstract]) OR obesity[Title/Abstract]) OR obese[Title/Abstract]) OR obeseness[Title/Abstract]	313.971

#14	Search (((Übergewicht[Title/Abstract]) OR Uebergewicht[Title/Abstract]) OR Fettleibigkeit*[Title/Abstract]) OR Fettsucht[Title/Abstract]	1
Table S2: Full search strategy PubMed – continued		
#15	Search #11 OR #12 OR #13 OR #14	357.087
#16	Search ""Primary Prevention""[Mesh]	147.496
#17	Search ""Health Promotion""[Mesh]	74.327
#18	Search ""School Health Services""[Mesh]	22.538
#19	Search ((primary prevention[Title/Abstract]) OR health promotion[Title/Abstract]) OR school health service*[Title/Abstract]	50.074
#20	Search (prevent*[Title/Abstract]) OR promot*[Title/Abstract]	2.236.781
#21	Search (((prävention[Title/Abstract]) OR praevention[Title/Abstract]) OR pravention[Title/Abstract]) OR Vorsorge*[Title/Abstract]	72
#22	Search #16 OR #17 OR #18 OR #19 OR #20 OR #21	2.401.349
#23	Search ""Body Weights and Measures""[Mesh]	589.099
#24	Search (((((BMI*) OR Body Mass ind*) OR Waist*) OR Hip*) OR Skin Fold*) OR (""Body Weights and Measures"")	440.016
#25	Search ((((Taille*) OR Huft*) OR Hüft*) OR Hueft*) OR Hautfalte*	2.920
#26	Search #23 OR #24 OR #25	867.668
#27	Search ((Program*[Title/Abstract]) OR Measure*[Title/Abstract]) OR Intervention*[Title/Abstract]	4.463.257
#28	Search ((Maßnahme[Title/Abstract]) OR Massnahme[Title/Abstract]) OR Masnahme[Title/Abstract]	2
#29	Search #27 OR #28	4.463.259
#30	Search #6 AND #10 AND #15 AND #22 AND #26 AND #29	2.528
#31	Search #6 AND #10 AND #15 AND #22 AND #26 AND #29 Filters: Publication date from 2015/01/01 to 2019/12/31	914
#32	Search #6 AND #10 AND #15 AND #22 AND #26 AND #29 Filters: Publication date from 2015/01/01 to 2019/12/31 English German	873

Table S3: Full search strategy Medline

Nr.	Search Term	Hits
#1	CHILD.af.	2.160.613
#2	ADOLESCENT.af.	2.041.462
#3	STUDENT?.af.	335.407
#4	(KIDS$ or CHILD??? or BOY? or GIRL? ORSTUDENT? or YOUTH? or TEEN???? or ADOLES#EN?).ab,ti.	1.644.929
#5	(JUNG?? or SCH??LER$ or KIND$ or MA??DCHEN or JUGEND or JUGENDALTER or JUGENDLICHE?).ab,ti.	176.258
#6	1 or 2 or 3 or 4 or 5	3.849.082
#7	SCHOOL?.af.	4.032.671
#8	(PRIMARY SCHOOL? or SECONDARY SCHOOL? or HIGH SCHOOL? or VOCATIONAL SCHOOL? or SCHOOLBASED or SCHOOL BASED or ELEMENTARY SCHOOL$ or GRAMMAR SCHOOL$ or HIGH SCHOOL$ or MIDDLE SCHOOL$ or COMPREHENSIVE SCHOOL$ or PUBLIC SCHOOL$ or INDEPENDENT SCHOOL$).ab,ti.	76.498
#9	(GRUNDSCHUL$ or REALSCHUL$ or HAUPTSCHUL$ or SCHULE? or SCHULISCHE or GYMNASI$ or PRIMARSTUFE? or SEKUNDARSTUFE? or SCHULBASIERT$ or GESAMTSCHUL$ or OBERSCHUL$).ab,ti.	668
#10	7 or 8 or 9	4.033.210
#11	OBESITY.af.	327.752
#12	ADIPOSITY.af.	28.779
#13	(ADIPOSITY or ADIPOSITAS or OVERWEIGHT or OBESITY or OBESE or OBESENESS).ab,ti.	308.340
#14	(U?BERGEWICHT or FETTLEIBIGKEIT?? or FETTSUCHT).ab,ti.	34
#15	11 or 12 or 13 or 14	373.974
#16	PRIMARY PREVENTION.af.	33.255
#17	HEALTH PROMOTION.af.	113.434
#18	SCHOOL HEALTH SERVICES.af.	17.043
#19	(PRIMARY PREVENTION or HEALTH PROMOTION or SCHOOL HEALTH SERVICE?).ab,ti.	46.353
#20	(PREVENT??? or PROMOT???).ab,ti.	2.184.005
#21	(PRA?VENTION or VORSORGE$).ab,ti.	228
#22	16 or 17 or 18 or 19 or 20 or 21	2.257.924
#23	('BODY WEIGHTS and MEASURES').af.	6.260
#24	(BMI$ or BODY MASS IND$ or WAIST$ or HIP$ or SKIN FOLD? or "BODY WEIGHTS AND MEASURES").af.	702.883
#25	(TAILLE$ or HU?FT$ or HAUTFALTE?).af.	6.910
#26	23 or 24 or 25	706.297
#27	(PROGRAM??? or MEASURE? or INTERVENTION??).ab,ti.	3.759.972
#28	MA??NAHME?.ab,ti.	374
#29	27 or 28	3.760.116
#30	6 and 10 and 15 and 22 and 26 and 29	3.358
#31	limit 30 to yr="2015 -Current"	1.522

Table S4: Full search strategy Embase

Nr.	Search Term	Hits
#1	'child'/exp	2.800.514
#2	'adolescent'/exp	1.615.769
#3	'student'/exp	249.373
#4	kids* OR child$$$ OR boy$ OR girl$ OR student$ OR youth$ OR teen$$$$ OR adoles?en$$:ab,ti	4.024.573
#5	jung$$ OR sch$$ler* OR kind* OR ma$$dchen OR jugend OR jugendalter OR jugendliche$:ab,ti	599.919
#6	#1 OR #2 OR #3 OR #4 OR #5	5.481.107
#7	'school' /exp	353.798
#8	'primary school$' OR 'secondary school$' OR 'high school$' OR 'vocational school$' OR schoolbased OR 'school based' OR 'elementary school*' OR 'grammar school*' OR 'high school*' OR 'middle school*' OR 'comprehensive school*' OR 'public school*' OR 'independent school*':ab,ti	110.282
#9	grundschul* OR realschul* OR hauptschul* OR schule$ OR schulische OR gymnasi* OR primarstufe$ OR sekundarstufe$ OR schulbasiert* OR gesamtschul* OR oberschul*:ab,ti	11.614
#10	#7 OR #8 OR #9	430.960
#11	'obesity'/exp	504.084
#12	'adiposity'/exp OR adiposity OR 'adipositas'/exp OR adipositas OR 'overweight'/exp OR overweight OR 'obesity'/exp OR obesity OR obese OR obeseness:ab,ti	626.432
#13	u$bergewicht OR fettleibigkeit$$ OR fettsucht:ab,ti	533
#14	#11 OR #12 OR #13	626.444
#15	'primary prevention'/exp	39.206
#16	'health promotion'/exp	96.017
#17	'school health service'/exp	23.065
#18	'primary prevention' OR 'health promotion' OR 'school health service$':ab,ti	191.314
#19	prevent$$$ OR promot$$$:ab,ti	4.098.817
#20	pra$vention OR vorsorge*:ab,ti	6.136
#21	#15 OR #16 OR #17 OR #18 OR #19 OR #20	4.162.856
#22	'morphometry'/exp	140.500
#23	bmi* OR 'body mass ind*' OR waist* OR hip* OR 'skin fold$' OR 'body weights and measures'/exp OR 'body weights and measures'	1.132.083
#24	taille* OR hu$ft* OR hautfalte$	8.297
#25	#22 OR #23 OR #24	1.136.039
#26	program$$$ OR measure$ OR intervention$$:ab,ti	5.604.495
#27	ma$$nahme$:ab,ti	80
#28	#26 OR #27	5.604.544
#29	#6 AND #10 AND #14 AND #21 AND #25 AND #28	2.445
#30	#6 AND #10 AND #14 AND #21 AND #25 AND #28 AND [2015-2019]/py	822
#31	#6 AND #10 AND #14 AND #21 AND #25 AND #28 AND [2015-2019]/py AND ([english]/lim OR [german]/lim)	800

Table S5: Full search strategy Cochrane library

#	Suche	Ergebnisse
1	MeSH descriptor: [Child] explode all trees	1.207
2	MeSH descriptor: [Adolescent] explode all trees	101.825
3	MeSH descriptor: [Students] explode all trees	4.140
4	(KIDS* OR CHILD* OR BOY? OR GIRL? OR STUDENT? OR YOUTH? OR TEEN* OR ADOLES?EN??):ti,ab,kw	185.789
5	(JUNG?? OR SCH??LER* OR KIND* OR MA??DCHEN OR JUGEND OR JUGENDALTER OR JUGENDLICHE?):ti,ab,kw	11.137
6	#1 OR #2 OR #3 OR #4 OR #5	259.812
7	MeSH descriptor: [Schools] explode all trees	2.843
8	(PRIMARY SCHOOL? OR SECONDARY SCHOOL? OR HIGH SCHOOL? OR VOCATIONAL SCHOOL? OR SCHOOLBASED OR SCHOOL BASED OR ELEMENTARY SCHOOL* OR GRAMMAR SCHOOL* OR HIGH SCHOOL* OR MIDDLE SCHOOL* OR COMPREHENSIVE SCHOOL* OR PUBLIC SCHOOL* OR INDEPENDENT SCHOOL*):ti,ab,kw	22.569
9	(GRUNDSCHUL* OR REALSCHUL* OR HAUPTSCHUL* OR SCHULE? OR SCHULISCHE OR GYMNASI* OR PRIMARSTUFE? OR SEKUNDARSTUFE? OR SCHULBASIERT* OR GESAMTSCHUL* OR OBERSCHUL*):ti,ab,kw	185
10	#7 OR #8 OR #9	23.831
11	MeSH descriptor: [Obesity] explode all trees	12.678
12	MeSH descriptor: [Adiposity] explode all trees	699
13	(ADIPOSITY OR ADIPOSITAS OR OVERWEIGHT OR OBESITY OR OBESE OR OBESENESS):ti,ab,kw	41.947
14	(U?BERGEWICHT OR FETTLEIBIGKEIT?? OR FETTSUCHT):ti,ab,kw	31
15	#11 OR #12 OR #13 OR #14	42.013
16	MeSH descriptor: [Primary Prevention] explode all trees	3.964
17	MeSH descriptor: [Health Promotion] explode all trees	6.053
18	MeSH descriptor: [School Health Services] explode all trees	1.492
19	(PRIMARY PREVENTION OR HEALTH PROMOTION OR SCHOOL HEALTH SERVICE?):ti,ab,kw	53.154
20	(PREVENT* OR PROMOT*):ti,ab,kw	245.686
21	(PRA?VENTION OR VORSORGE*):ti,ab,kw	171
22	#16 OR #17 OR #18 OR #19 OR #20 OR #21	248.342
23	"BODY WEIGHT AND MEASURES"	2
24	BMI* OR BODY MASS IND* OR WAIST* OR HIP* OR SKIN FOLD? OR "BODY WEIGHTS AND MEASURES"	94.517
25	TAILLE* OR HU?FT* OR HAUTFALTE?	1.438
26	#23 OR #24 OR #25	95.227
27	(PROGRAM??? OR MEASURE? OR INTERVENTION??):ti,ab,kw	589.974
28	(MA??NAHME?):ti,ab,kw	266
29	#27 OR #28	589.990
30	#6 AND #10 AND #15 AND #22 AND #26 AND #29	756
31	#6 AND #10 AND #15 AND #22 AND #26 AND #29<br>with Publication Year from 2015 to 2019	461

Table S6: Full search strategy BIOSIS Preview

#	Suche	Ergebnisse
1	CHILD.af.	526.246
2	ADOLESCENT.af.	142.932
3	STUDENT?.af.	95.677
4	(KIDS$ Or CHILD??? or BOY? or GIRL? or STUDENT? or YOUTH? or TEEN???? or ADOLES#EN??).ab,ti.	779.037
5	(JUNG?? or SCH??LER$ or KIND$ or MA??DCHEN or JUGEND or JUGENDALTER or JUGENDLICHE?).ab,ti.	176.102
6	1 or 2 or 3 or 4 or 5	1.208.791
7	SCHOOL?.af.	711.393
8	(PRIMARY SCHOOL? or SECUNDARY SCHOOL? or HIGH SCHOOL? or VOCATIONAL SCHOOL? or SCHOOLBASED or SCHOOL BASED or ELEMENTARY SCHOOL$ or GRAMMAR SCHOOL$ or HIGH SCHOOL$ or MIDDLE SCHOOL$ or COMPREHENSIVE SCHOOL$ or PUBLIC SCHOOL$ or INDEPENDET SCHOOL$).ab,ti.	23.634
9	(GRUNDSCHUL$ or REALSCHUL$ or HAUPTSCHUL$ or SCHULE? or SCHULISCHE or GYMNASI$ or PRIMARSTUFE? or SEKUNDARSTUFE? or SCHULBASIERT$ or GESAMTSCHUL$ or OBERSCHUL$).ab,ti.	422
10	7 or 8 or 9	711.741
11	OBESITY.af.	302.252
12	ADIPOSITY.af.	7.133
13	(ADIPOSITY or ADIPOSITAS or OVERWEIGHT or OBESITY or OBESE or OBESENESS).ab,ti.	99.146
14	(U?BERGEWICHT or FETTLEIBIGKEIT?? or FETTSUCHT).ab,ti.	101
15	11 or 12 or 13 or 14	318.237
16	PRIMARY PREVENTION.af.	5.003
17	HEALTH PROMOTION.af.	7.387
18	SCHOOL HEALTH SERVICES.af.	158
19	(PRIMARY PREVENTION or HEALTH PROMOTION or SCHOOL HEALTH SERVICE?).ab,ti.	7.997
20	(PREVENT??? or PROMOT???).ab,ti.	1.190.682
21	(PRA?VENTION or VORSORGE$).ab,ti.	129
22	16 or 17 or 18 or 19 or 20 or 21	451.175
23	“BODY WEIGHTS AND MEASURES”.af.	4
24	(BMI$ or BODY MASS IND$ or WAIST$ or HIP$ or SKIN FOLD? or “BODY WEIGHTS AND MEASURES”).af.	219.807
25	(TAILLE$ or HU?FT$ or HAUTFALTE?).af.	2.314
26	23 or 24 or 25	321.895
27	(PROGRAM??? or MEASURE? or INTERVENTION??).ab,ti.	2.176.366
28	MA??NAHME?.ab,ti.	200
29	27 or 28	2.176.455
30	6 and 10 and 15 and 22 and 26 and 29	174
31	30 and PY>=2015	0

Table S7: Full search strategy DAHTA

Nr.	Suchformulierung	Trefferzahl
41	12 AND 20 AND 26 AND 33 AND 37 AND 40	0
40	38 OR 39	262
39	FT=MA##NAHME#	81
38	(FT=PROGRAM### OR FT=MEASURE#) OR FT=INTERVENTION##	244
37	34 OR 35 OR 36	18
36	(FT=TAILLE? OR FT=HU#FT?) OR FT=HAUTFALTE#	11
35	(((FT=BMI? OR FT=BODY MASS IND?) OR FT=WAIST?) OR FT=HIP?) OR FT=SKIN FOLD#	14
34	FT=BODY WEIGHTS "AND" MEASURES	0
33	27 OR 28 OR 29 OR 30 OR 31 OR 32	141
32	FT=PRA#VENTION OR FT=VORSORGE?	73
31	FT=PREVENT### OR FT=PROMOT###	137
30	(FT=PRIMARY PREVENTION OR FT=HEALTH PROMOTION) OR FT=SCHOOL HEALTH SERVICE#	30
29	FT=SCHOOL HEALTH SERVICES	0
28	FT=HEALTH PROMOTION	3
27	FT=PRIMARY PREVENTION	30
26	21 OR 22 OR 23 OR 24 OR 25	14
25	(FT=U#BERGEWICHT OR FT=FETTLEIBIGKEIT##) OR FT=FETTSUCHT	10
24	FT=OBESENESS	0
23	(((FT=ADIPOSITY OR FT=ADIPOSITAS) OR FT=OVERWEIGHT) OR FT=OBESITY) OR FT=OBESE	13
22	FT=ADIPOSITY	6
21	FT=OBESITY	8
20	13 OR 14 OR 15 OR 16 OR 17 OR 18 OR 19	12
19	FT=OBERSCHUL?	0
18	(((FT=GYMNASI? OR FT=PRIMARSTUFE#) OR FT=SEKUNDARSTUFE#) OR FT=SCHULBASIERT?) OR FT=GESAMTSCHUL?	1
17	(((FT=GRUNDSCHUL? OR FT=REALSCHUL?) OR FT=HAUPTSCHUL?) OR FT=SCHULE#) OR FT=SCHULISCHE	4
16	(FT=COMREHENSIVE SCHOOL? OR FT=PUBLIC SCHOOL?) OR FT=INDEPENDENT SCHOOL?	0
15	(((FT=SCHOOL BASED OR FT=ELEMENTARY SCHOOL?) OR FT=GRAMMAR SCHOOL?) OR FT=HIGH SCHOOL?) OR FT=MIDDLE SCHOOL?	2
14	(((FT=PRIMARY SCHOOL# OR FT=SECONDARY SCHOOL#) OR FT=HIGH SCHOOL#) OR FT=VOCATIONAL SCHOOL#) OR FT=SCHOOLBASED	0
13	FT=SCHOOL#	12
12	3 OR 4 OR 6 OR 8 OR 9 OR 10 OR 11	75
11	FT=JUGENDALTER OR FT=JUGENDLICHE#	20
10	(((FT=JUNG## OR FT=SCH##LER?) OR FT=KIND?) OR FT=MA##DCHEN) OR FT=JUGEND	71
9	(FT=YOUTH# OR FT=TEEN####) OR FT=ADOLES%EN##	19
8	(((FT=KIDS? OR FT=CHILD###) OR FT=BOY#) OR FT=GIRL#) OR FT=STUDENT#	42
6	FT=STUDENT#	1
4	FT=ADOLESCENT	10
3	FT=CHILD	28
1	Datenbankauswahl: DAHTA	704

Table S8: Full search strategy DARE & NHSEED

Nr.	Suchformulierung	Treffer
1	MeSH DESCRIPTOR child EXPLODE ALL TREES IN DARE,NHSEED	4.363
2	MeSH DESCRIPTOR adolescent EXPLODE ALL TREES IN DARE,NHSEED	4.368
3	MeSH DESCRIPTOR students EXPLODE ALL TREES IN DARE,NHSEED	86
4	(kids* OR child* OR boy* OR girl* OR student* OR youth* OR teen* OR adolescen* OR adoleszen*) IN DARE, NHSEED	11.116
5	(jung* OR schuler* OR schueler* OR kind* OR madchen OR maedchen OR jugend OR jugendalter OR jugendliche*) IN DARE, NHSEED	498
6	#1 OR #2 OR #3 OR #4 OR #5	11.461
7	MeSH DESCRIPTOR Schools EXPLODE ALL TREES IN DARE,NHSEED	173
8	(primary school* OR secondary school* OR high school* OR vocational school* OR schoolbased OR school based OR elementary school* OR grammar school* OR middle school* OR comprehensive school* OR public school* OR independent school*) IN DARE, NHSEED	383
9	(grundschul* OR realschul* OR hauptschul* OR schule* OR schulische OR gymnasi* OR primarstufe* OR sekundarstufe* OR schulbasiert* OR gesamtschul* OR oberschul*) IN DARE, NHSEED	32
10	#7 OR #8 OR #9	506
11	MeSH DESCRIPTOR Obesity EXPLODE ALL TREES IN DARE,NHSEED	834
12	MeSH DESCRIPTOR adiposity EXPLODE ALL TREES IN DARE,NHSEED	19
13	(adiposity OR adipositas OR overweight OR obesity OR obese OR obeseness) IN DARE, NHSEED	1.348
14	(Ubergewicht OR uebergewicht OR fettleibigkeit* OR fettsucht) IN DARE, NHSEED	1
15	#11 OR #12 OR #13 OR #14	1.352
16	MeSH DESCRIPTOR primary prevention EXPLODE ALL TREES IN DARE,NHSEED	809
17	MeSH DESCRIPTOR health promotion EXPLODE ALL TREES IN DARE,NHSEED	811
18	MeSH DESCRIPTOR school health services EXPLODE ALL TREES IN DARE,NHSEED	159
19	(primary prevention OR health promotion OR school health service*) IN DARE, NHSEED	2.383
20	(prevent* OR promot*) IN DARE, NHSEED	17.052
21	(pravention OR praevention OR vorsorge*) IN DARE, NHSEED	2
22	#16 OR #17 OR #18 OR #19 OR #20 OR #21	17.148
23	(bmi* OR body mass ind* OR waist* OR hip* OR skin fold* OR body weight measures) IN DARE, NHSEED	2.302
24	(taille* or hueft* OR huft* OR hautfalte) IN DARE, NHSEED	7
25	#23 OR #24	2.307
26	(program* OR measure* OR intervention*) IN DARE, NHSEED	29.653
27	(massnahme* OR maßnahme* OR masnahme*) IN DARE, NHSEED	0
28	#26 OR #27	29.653
29	#6 AND #10 AND #15 AND #22 AND #25 AND #28	50
30	(#6 AND #10 AND #15 AND #22 AND #25 AND #28) IN DARE, NHSEED FROM 2015 TO 2019	0

Table S9: Full search strategy HTA (INAHTA)

Nr.	Suchformulierung	Treffer
1	MeSH DESCRIPTOR child EXPLODE ALL TREES IN DARE,NHSEED	572
2	MeSH DESCRIPTOR adolescent EXPLODE ALL TREES IN DARE,NHSEED	226
3	MeSH DESCRIPTOR students EXPLODE ALL TREES IN DARE,NHSEED	2
4	(kids* OR child* OR boy* OR girl* OR student* OR youth* OR teen* OR adolescen* OR adoleszen*) IN DARE, NHSEED	1.390
5	(jung* OR schuler* OR schueler* OR kind* OR madchen OR maedchen OR jugend OR jugendalter OR jugendliche*) IN DARE, NHSEED	145
6	#1 OR #2 OR #3 OR #4 OR #5	1.484
7	MeSH DESCRIPTOR Schools EXPLODE ALL TREES IN DARE,NHSEED	27
8	(primary school* OR secondary school* OR high school* OR vocational school* OR schoolbased OR school based OR elementary school* OR grammar school* OR middle school* OR comprehensive school* OR public school* OR independent school*) IN DARE, NHSEED	46
9	(grundschul* OR realschul* OR hauptschul* OR schule* OR schulische OR gymnasi* OR primarstufe* OR sekundarstufe* OR schulbasiert* OR gesamtschul* OR oberschul*) IN DARE, NHSEED	17
10	#7 OR #8 OR #9	79
11	MeSH DESCRIPTOR Obesity EXPLODE ALL TREES IN DARE,NHSEED	191
12	MeSH DESCRIPTOR adiposity EXPLODE ALL TREES IN DARE,NHSEED	1
13	(adiposity OR adipositas OR overweight OR obesity OR obese OR obeseness) IN DARE, NHSEED	277
14	(Ubergewicht OR uebergewicht OR fettleibigkeit* OR fettsucht) IN DARE, NHSEED	1
15	#11 OR #12 OR #13 OR #14	278
16	MeSH DESCRIPTOR primary prevention EXPLODE ALL TREES IN DARE,NHSEED	105
17	MeSH DESCRIPTOR health promotion EXPLODE ALL TREES IN DARE,NHSEED	78
18	MeSH DESCRIPTOR school health services EXPLODE ALL TREES IN DARE,NHSEED	10
19	(primary prevention OR health promotion OR school health service*) IN DARE, NHSEED	204
20	(prevent* OR promot*) IN DARE, NHSEED	2.326
21	(pravention OR praevention OR vorsorge*) IN DARE, NHSEED	21
22	#16 OR #17 OR #18 OR #19 OR #20 OR #21	2.377
23	(bmi* OR body mass ind* OR waist* OR hip* OR skin fold* OR body weight measures) IN DARE, NHSEED	389
24	(taille* or hueft* OR huft* OR hautfalte) IN DARE, NHSEED	0
25	#23 OR #24	389
26	(program* OR measure* OR intervention*) IN DARE, NHSEED	5.274
27	(massnahme* OR maßnahme* OR masnahme*) IN DARE, NHSEED	5
28	#26 OR #27	5.274
29	#6 AND #10 AND #15 AND #22 AND #25 AND #28	4
30	(#6 AND #10 AND #15 AND #22 AND #25 AND #28) IN DARE, NHSEED FROM 2015 TO 2019	0
