# Supplementary material for: Waist circumference as a parameter in school-based interventions to prevent overweight and obesity - a systematic review and meta-analysis
Source: BMC Public Health. 2024 Oct 17;24:2864. doi: 10.1186/s12889-024-20354-7 (PMC11488270; doi:10.1186/s12889-024-20354-7)
Supplement: Supplementary file 2 — Supplementary Material 2: Additional file 2. Design and characteristics – randomised controlled trials. [file 12889_2024_20354_MOESM2_ESM.pdf]

Kula et al. Additional file 2. Design and characteristics – randomised controlled trials: included studies sorted alphabetically

**Included studies sorted alphabetically: randomised controlled trials**			
First author & publication year, program name, country of origin, study design specifics	Sample / Participants	Key components of intervention	Main results regarding waist circumference
**Indiviudal randomised controlled trials**			
Rerksuppaphol 2017[67], nn, Central Thailand	Public elementary schools<br>1st to 6th grade students<br>Age (years) mean (SD):<br>IG 10.2 (3.1); CG 10.0 (3.1)<br>Participants at baseline:<br>All n = 217; IG n = 111; CG n= 106<br>Proportion female at baseline:<br>IG 53.2%; CG 49.1%	• Internetbased program<br>• HE focused on diet and PA<br>• Self-monitoring component<br>• Monthly individual feedback plus recommendations<br>• Promotion of at leat 60 min PA per day<br>(age-appropriate examples were given)<br>Intervention period: 4 months<br>***CG:** after baseline assessment: individual nutritional status was interpreted and informed knowledge of proper healthy diet, PA and sedentary behaviors was advised by trained research assistants*	No effect estimate reported for WC<br>WC (cm) Median (IQR)<br>*Baseline*<br>IG 63.5 (56.0 to 72)<br>CG 65.0 (56 to 77)<br>*Posttest*<br>IG 67 (59 to.73)<br>CG 68 (59 to 79.1)
**Cluster randomised controlled trials**			
Adab 2018[50],<br>WAVES, UK<br><br>block randomisation (SES variables, ethnic variables; school size)<br><br>Unit of randomisation: school	Primary schools:<br>IG n = 26; CG n = 28<br>1st grade students<br>Age (years) mean (SD):<br>6.3 (0.3)<br>Participants at baseline:<br>All n = 1397; IG n = 662; CG n = 735<br>Proportion female at baseline:<br>All 48.9%; IG 50.8%; CG 47.3%	• Teacher training<br>• Additional PA delivered by class teacher (30 min per school day in units of 5 to 15 minutes in class or at recess)<br>• Promotion of development of a healthy lifestyle ('Villa Vitality event' with iconic sports institution and interactive session on healthy eating and healthy lifestyle, one block focusing on PA, one block focusing on diet, designed as class project including family activities)<br>• Cooking skills workshops, parents were offered to take part<br>• Parent HE sessions on diet<br>• Information for families on local leisure opportunities<br>Intervention period: 1 academic year<br>***CG:** additional citizenship education resources excluding topics related to healthy eating or PA*	WC z-score<br>Difference in mean change (99% CI)<br>IG vs CG<br>*Posttest*<br>Adjusted for baseline value:<br>0.026 (-0.229; 0.281) p=0.794<br>Further adjusted (students' covariates):<br>0.019 (-0.166; 0.205) p=0.789<br>*FU 18 months after end of intervention:*<br>Adjusted for baseline value:<br>0.103 (-0.087; 0.293) p=0.163<br>Further adjusted (students' covariates):<br>0.068 (-0.133; 0.269) p=0.383<br>Cluster design considered
Brandstetter 2012[75],<br>URMEL-ICE, Germany<br><br>stratified randomisation (school size, demographic variables)<br>Unit of randomisation:	Primary schools:<br>IG n = 16; CG n = 16<br>2nd grade students<br>Age (years) mean (SD):<br>IG 7.61 (0,42); CG 7.53 (0,42)<br>Participants at baseline:<br>All n = 1119; IG n = 540; CG n = 579	• Teacher training<br>• HE focused on sugar-sweetened beverages, screen time and PA<br>(regular teaching units focused on health-promotion behavior change)<br>• Short blocks of PA of 5-7 minutes twice a schoolday<br>• Family homework lessons (children need help of family members to solve the tasks, six times)<br>Intervention period: 1 academic year	WC (cm)<br>Regression Coefficient β (95% CI)<br>IG vs CG<br>Adjusted for baseline value<br>- 0.85 (-1.59; -0.12)<br>*regression coefficients correspond to the differences between CG and IG*

school	Proportion female at baseline:<br>IG 44.9%; CG 47.9%		Cluster design considered
Brito Beck da Silva 2019[51],<br>Stayingfit Brazil, Brazil<br><br>Unit of randomisation:<br>school	Midsized public schools:<br>IG n = 16; CG n = 16<br>7th to 9th grade students<br>Age (years) mean (SD):<br>All 14.49 (1.42)<br>Participants at baseline:<br>All n = 895; IG n = 428; CG n = 467<br>Proportion female at baseline:<br>All 48.4%; IG 46.0%; CG 50.5%	• HE on healthy lifestyle, diet, PA, body image and behavior change<br>• Online tool<br>○ Accessed indiviudally once a week<br>○ Including personal PA log, Food Log and Goals Log<br>○ Support of nutrionist and assistant<br>• Parent material on subjects of HE units<br>• Additional material for teachers<br>Intervention period: 1 academic year	No effect estimate reported for WC<br>WC (cm)<br>Difference over time<br>IG 1.11 (analyzed n=285)<br>CG 2.00 (analyzed n=314)<br>Diff IG vs Diff CG p=0.393<br>Cluster design considered
Christiansen 2013[77,]<br>SPACE, Denmark<br><br>schools match paired (SES variables, demographics, distance of residents from school, urbanity, school outdoor area)<br><br>Unit of randomisation:<br>school	Sample schools:<br>IG n = 7; CG n = 7<br>5th and 6th grade students<br>Age (years) mean (SD):<br>IG 12.6 (0.6); CG 12.6 (0.6)<br>Participants at baseline:<br>All n = 1348; IG n = 623; CG n = 725<br>Proportion female at baseline:<br>IG 49.3%, CG 47.7%	• Restructured school outdoor area for PA according to a catalogue of 11 prioritized components (including unfixed equipment)<br>• Improvement of safety for active commuting<br>(school's traffic patrol, safe cycling courses)<br>• School theme week focusing on PA during school lessons<br>• Promoting PA during breaks/recess<br>(facilitated by trained teachers and older students)<br>• Formulation and implementation of school PA policy<br>• Financial support for adjustments<br>• Organizational support (one and a half full-time positions for coordination, development and coordination)<br>Intervention period: 1 academic year	WC (cm)<br>Difference in mean change (95% CI)<br>IG vs CG<br>Adjusted for age, sex, baseline value<br>0,2 (-2.5; 2.8); p=0.91; ICC=0.22<br>Cluster design considered
Eather 2015[53],<br>CrossFit Teens™, Australia<br><br>Unit of randomisation:<br>class	Secondary school: n = 1)<br>Classes: n = 4<br>10th grade students<br>Age (years) mean (SD):<br>15.5 (0.5)<br>Participants at baseline:<br>All n = 96; IG n = 51; CG n = 45<br>Proportion female at baseline:<br>All 52%	• Restructured PE and sports lesson (CrossFit Traininig), delivered by experienced CrossFit instructors<br>Intervention period: 8 weeks	WC (cm)<br>Difference in mean change (95% CI)<br>IG vs CG<br>Adjusted for baseline scores<br>-3.1 (-5.00; -1.25) p<0.001<br>Cohen's *d*: 0.2<br>Cluster design considered
Farias 2015[55],<br>nn, Brazil<br><br>Unit of randomisation:<br>class	High school: n = 1<br>Classes: IG n=5, CG n=5<br>1st to 3rd year high school students<br>Age (years) mean (SD):<br>IG 15.9 (0.8), CG 16.0 (0.8)<br><br>Participants at baseline:<br>All n = 286; IG n = 195; CG n = 191<br>Proportion female at baseline:	• Restructured PE lessons with heart rate monitoring<br>(aerobic activity, sports games, stretching)<br>Intervention period: 1 academic year	WC (cm)<br>Difference over time (SE)<br>*Boys:*<br>IG -2.42 (0.19); CG 0.86 (0.12)<br>IG vs CG p<0.001<br><br>*Girls:*<br>IG -2.21 (0.14); CG 0.64 (0.12)<br>IG vs CG p<0.001

	IG 43.1%; CG 50.7%		
Farmer 2017[56],<br>PLAY, New Zealand<br><br>schools match paired (region, size of school, SES variables)<br><br>Unit of randomisation: school	State primary schools:<br>IG n = 8; CG n = 8<br>2nd and 4th grade students<br>Age (years) mean (SD):<br>IG 8.0 (1.2), CG 7.9 (1.1)<br>Participants at baseline:<br>All n = 840; IG n = 418; CG n = 422<br>Proportion female at baseline:<br>IG 47.2%; CG 52.9%	• Development of a playground action plan (researchers, playworkers and school community)<br>• Restructred playground environment<br>(including interactive equipment)<br>• Change in school rules and policies<br>• Provision of start-up fund of NZDs 15.000<br>Intervention period: 2 academic years	WC (cm)<br>Regression Coefficient β (95% CI)<br>Adjusted for age, sex, baseline value<br>IG vs CG<br>-0.4 (-1.1; 0.4) p=0.325<br>*regression coefficient corresponds to the difference between CG and IG*<br>Cluster design considered
Foster 2010[78],<br>HEALTHY, USA<br><br>Unit of randomisation: school	Sample schools:<br>≥ 50% students subsidised lunch or ≥ 50% Black/Hispanic students<br>IG n = 21; CG n = 21<br>6th grade students<br>Age (years) mean (SD):<br>All 11.3 (0.6)<br>Participants at baseline:<br>All n = 209; IG n = 103; CG n = 106<br>Proportion female (analyzed):<br>All 52.7%; IG 52.6%; CG 52.9%	• Restructured school food environment (including snack bars, school stores, vending machines, classroom parties and celebrations)<br>• Cafeteria laboratories and taste test events (interactive learning on health promoting changes for students)<br>• Restructured PE lessons<br>• HE on nutrition, PA and lifestyle (included in various topics, encompassing behavior change activities, regularly over the intervention period)<br>○ Teacher manual<br>• Newsletter to caregivers and families<br>Intervention period: 2.5 academic years	No effect estimate reported for WC<br>WC (cm) mean (SD)<br>*Baseline*<br>IG 76.0 (15.1); CG 75.7 (14.8)<br>*Posttest*<br>IG 80.6 (14.8); CG 81.0 (14.8)<br>WC (cm) Difference over time<br>IG 4.6; CG 5.3<br>IG vs CG p=0.07<br>Cluster design considered
Grydeland 2013[79],<br>HEIA, Norway<br><br>Unit of randomisation: school	Schools > 40 students in 6th grade in large towns:<br>IG n = 12; CG n = 25<br>6th grade students<br>Age (years) mean (SD):<br>IG 11.2 (0.39); CG 11.2 (0.3)<br>Participants at baseline:<br>All 1485; IG n = 527; CG n = 958<br>Proportion female at baseline:<br>IG 50%; CG 48%	• HE on diet, PA and screen time<br>○ Classroom units in first intervention year<br>○ Computer tailored individual advice in second intervention year<br>○ Teacher booklet<br>• Fruit and vegetable break in class once a week (Cutting equipment provided)<br>• Provision of Info folder and resource box with equipment for cutting and selling Fruits and Vegetables (once for 7th grade)<br>• PA breaks in regular class (10 min once a week)<br>• Provision of sports equipment for recess/breaks<br>• Active commuting campaigns<br>• Info Material for caregivers/families<br>Intervention period: 2 academic years	No effect estimate reported for WC<br>WC (cm) mean (SD)<br>*Baseline*<br>IG 62.7 (6.1); CG 63.3 (6.5)<br>WC (cm) mean (95% CI)<br>*Posttest*<br>IG 66.4 (66.0; 66.7);<br>CG 66.2 (66.0;66.5.)<br>No statistical significant effect for WC<br>Cluster design considered
Habib-Mourad 2014[80]<br>Health-E-PALs, Lebanon<br><br>schools match paired	Private and public schools<br>IG n = 4; CG n = 4<br>4th and 5th grade students<br>Age (years) mean (SD):	• HE on diet, PA and screen time<br>(classroom lessons once per week)<br>• Improvement of food service targeting school shops and	WC (cm)<br>No statistical significant change for WC<br>p>0.05 in each group

(SES variales, religious sect)<br><br>Unit of randomisation: school	IG 10.3 (0.9); CG 10.1 (1.0)<br>Participants at baseline:<br>All n = 374; IG n = 193; CG n = 181<br>Proportion female at baseline:<br>All 45.5%; IG 43.0%; CG 47.0%	• lunch boxes<br>• Family program (meetings, health fairs, information packages)<br>Intervention period: 3 months	Cluster design considered
Jansen 2011[82],<br>Lekker Fit!, Netherlands<br><br>schools match paired (school size, demographic variables)<br><br>Unit of randomisation: school	Primary schools:<br>IG n = 10; CG n = 10<br>*3rd to 5th grade students*<br>Age (years) mean (SD):<br>IG 7.7 (1.0); CG 7.8 (1.0)<br>Proportion female at baseline:<br>IG 50.5%; CG 51.0%<br>*6th to 8th grade students*<br>Age (years) mean (SD):<br>IG 10.8 (1.0); CG 10.8 (1.0)<br>Proportion female at baseline:<br>IG 52.8%; CG 49%<br>Participants at baseline:<br>All n = 2622; IG n = 1271; CG n = 1499	• Restructured PE lessons by special PE teachers<br>(funded by Municipal sports department)<br>• Voluntary sport and play activities outside school hours<br>• Involvement of local sports clubs<br>• HE on diet, active living and healthy lifestyle choices (adapted for each grade)<br>• Health promotion gathering for caregivers at the beginning<br>*Additional component for students with overweight/obesity: Individual counselling by school nurse if needed after assessments*<br>Intervention period: 1 academic year	WC (cm)<br>Regression Coefficient β (95% CI)<br>IG vs CG<br>3rd to 5th grade students<br>-1.29 (-2.16; -0.42)<br>*6th to 8th grade students*<br>-0.71 (-1.72; 0.29)<br>*regression coefficient corresponds to the difference between CG and IG*<br>Cluster design considered
Kipping 2014[84],<br>AFLY 5, UK<br><br>stratified randomisation (SES variables, school involvement in health promotion initiatives)<br><br>Unit of randomisation: school	Primary schools:<br>IG n = 30; CG n = 30<br>4th grade students<br>Age (years) mean (SD):<br>IG 9.5 (0.3); CG 9.5 (0.3)<br>Participants at baseline:<br>All n = 1842; IG n = 889; CG n = 953<br>Proportion female at baseline:<br>IG 49%; CG 52%	• Teacher / Learning support staff training<br>• HE on diet, PA, screen time<br>○ 16 teaching units<br>○ Provision of lesson plans and material<br>○ Provision of 10 caregiver-child interactive homework acitivities<br>• Schools were financially compensated for cost of replacement of teachers during teacher training<br>• Provison of written information for school newsletters<br>• Provision of written information for caregivers<br>Intervention period: 1 academic year	WC z-score<br>Difference in mean change (95% CI)<br>IG vs CG<br>Adjusted for age,sex,baseline value, stratification variables<br>-0.12 (-0.23; -0.01) p=0.03<br>Cluster design considered
Kocken 2016[58],<br>Extra Fit!, Netherlands<br><br>schools match paired (SES variables, educational level, urbanization)<br>Unit of randomisation: school	Primary schools:<br>IG n = 23; CG n = 22<br>9 to 11 years old students<br>Age (years) mean (SD):<br>IG 9.2 (0.6); CG 9.1 (0.6)<br><br>Participants at baseline:<br>All n = 1112; IG n = 615; CG n = 497<br>Proportion female at baseline:<br>IG 52.0%; CG 51.3%	• HE focused on diet and PA<br>○ Including practical exercises (diet and PA)<br>• Program structured according to the theory of planned behavior and targeted knowledge, attitude, social norms and perceived behavioral control<br><br>• Promotion of teacher and caregiver involvement by family events<br>Intervention period: 2 academic years	WC z-score<br>Difference in mean change (95% CI)<br>Adjusted for age, sex, baseline value, SES, ethnic variables<br>IG vs CG<br>-0.03 (-0.24; 0.17)<br>Cluster design considered
Kriemler 2010[85],	Sample schools: IG n = 9; CG n = 6	• Daily PE lessons	WC z-score

KISS, Switzerland<br><br>IG and CG schools located in provinces with similar SES characteristics<br><br>Unit of randomisation: school	Classes: IG n = 16, CG n = 12<br>1st and 5th grade students<br>Age (years) mean (SD):<br>1st grade: IG 6.9(0.3); CG 6.9(0.3)<br>5th grade: IG 11.0(0.5); CG 11.3(0.6)<br>Participants at baseline:<br>All n = 502; IG n = 297; CG n = 205<br>Proportion female at baseline:<br>1st grade: IG 49%; CG 45%<br>5th grade: IG 45%; CG 46%	○ Three times a week with specified curriculum by classroom teachers<br>○ Twice a week mostly outdoors, by PE teachers<br>• Short PA breaks during academic lessons (2-5 min, daily)<br>• PA homework (10 min, daily)<br>Intervention period: 1 academic year	Difference in average change (95% CI)<br>Adjusted for grade, sex, baseline value<br>IG vs CG<br>-0.08 (-0.2; 0.05) p=0.25 ICC 0.17<br>Cluster design considered
Leme 2016[59,60],<br>Healthy Habits, Healthy Girls Brazil, Brazil<br>*Girls*<br><br>schools match paired (geographicel location, school size, demographics)<br><br>Unit of randomisation: school	Technical schools (public secondary schools) that offer nutrition and dietetic technical courses:<br>IG n = 5; CG n = 5<br>7th to 9th grade female students<br>Age range: 14 to 18 years<br>Participants at baseline:<br>All n = 253; IG n = 142; CG n = 111<br>Proportion female at baseline:<br>Exclusively Girls	• Cultural adaptation of NEAT Girls<br>(The Nutrition Enjoyable Acitivity for Teen Girls)<br>• Teacher training<br>• HE on diet and PA (including nutrition and PA handbooks)<br>• Enhanced PE sessions (trained PE teachers)<br>• Seminar and nutrition workshops (dietitians)<br>• Social campaign using posters and messengers<br>• Diet and PA diaries for self-monitoring<br>• Newsletters for caregivers<br>Intervention period: 6 months	WC (cm) *Girls*<br>Difference in mean change (SE)<br>IG vs CG<br>-2.28(0.77)* p=0.014<br>Cluster design considered
Lloyd 2012[86],<br>HeLP, UK<br><br>Unit of randomisation: school	Primary or junior schools in Exeter:<br>IG n = 2; CG n = 2<br>5th grade students<br>Age (years) mean (SD):<br>IG 9.69 (0.3); CG 9.69 (0.3)<br>Participants at baseline:<br>All n = 202; IG n = 80; CG n = 122<br>Proportion female at baseline:<br>All 50%; IG 50%; CG 50%	Spring term:<br>• HE focusing on diet and PA<br>• Whole school assembly<br>• Newsletter articles<br>• Two activity workhops by professional sportsmen/dancers (caregivers can observe)<br>• Parents evening including child perfomances<br>• Support by program coordinators<br>Summer term:<br>• Healthy life styles week<br>• Promoting behavior change focused on goal setting<br>○ Questionnaire<br>○ Goal setting sheet, involving caregivers<br>• Parent evening<br>• Supported by program coordinators<br>Autumn term:<br>• Reinforcement activities<br>• Newsletter articles<br>• Whole school assembly	WC (cm)<br>Difference in mean change (95% CI)<br>IG vs CG<br>-2.01 (-9.54; 5.52)<br><br>WC z-score<br>Difference in mean change (95% CI)<br>IG vs CG<br>-0,32 (-1,52; 0,87)<br>Cluster design considered

		• Goal supporting interview<br>• HE lessons<br>• Support by program coordinators)<br>Intervention period: 1 academic year	
Lloyd 2018[61],<br>HeLP, UK<br><br>stratified randomisation (SES variable, one versus more than one year-5 class)<br><br>Unit of randomisation: school	Primary or junior schools:<br>IG n = 16; CG n = 16<br>5th grade students<br>Age (years) mean (SD):<br>IG 9,8 (0,3); CG 9,7 (0,3)<br>Participants at baseline:<br>All n = 1324; IG n = 676; CG n = 648<br>Proportion female at baseline:<br>IG 50%; CG 53%	Spring term:<br>• HE focusing on diet and PA<br>• Whole school assembly<br>• Newsletter articles<br>• Two activity workhops by professional sportsmen/dancers (caregivers can observe)<br>• Parents evening including child perfomances<br>• Support by program coordinators<br>Summer term:<br>• Healthy life styles week<br>• Promoting behavior change focused on goal setting<br>○ Questionnaire<br>○ Goal setting sheet, involving caregivers<br>• Parent evening<br>• Supported by program coordinators<br>Autumn term:<br>• Reinforcement activities<br>• Newsletter articles<br>• Whole school assembly<br>• Goal supporting interview<br>• HE lessons<br>• Support by program coordinators<br>Intervention period: 1 academic year	WC SD-score<br>Difference in mean change (95% CI)<br>IG vs CG<br>Adjusted for age, sex, baseline value, stratification variables<br>0.07 (-0.27; 0.12)<br>Cluster design considered
Lubans 2011[87],<br>PALs, Australia<br>*Boys*<br><br>Unit of randomisation: school	Secondary schools in low SES areas:<br>IG n = 2; CG n = 2<br>9th grade male students, considered by PE teachers to be disengaged in PE and/or not currently participating in team or individual sports<br>Age (years) mean (SD):<br>All 14.3 (0.6)<br>Participants at baseline:<br>All n = 100; IG n = 50; CG n = 50<br>Proportion female at baseline:<br>0%, exclusively Boys	• Restructured PE lessons<br>• Lunch-time PA sessions<br>• HE on diet and PA<br>(workbooks with information and home challenges for students and caregivers)<br>• PA leadership sessions<br>(participants recruited and instructed lower grade students in safely using elastic tubing resistance training devices)<br>• Pedometer and sessions on goal setting and behavior change<br>Intervention period: 6 months	WC (cm) *Boys*<br>Difference in mean change (95% CI)<br>Adjusted for covariates<br>IG vs CG<br>0.8 (-0.6; 2.3) p=0.23 Cohen's *d* 0.2<br>Cluster design considered

Lubans 2016[62],<br>ATLAS, Australia<br>*Boys*<br><br>schools match paired (geographical location, school size, demographics)<br><br>Unit of randomisation: school	Secondary schools in low income areas:<br>IG n = 7; CG n = 7<br>1st grade male students in secondary school, with < 60 min MVPA/day or more than 2hours screen-time/day;<br>Age (years) mean (SD):<br>All 12.7 (0.5)<br>Participants at baseline:<br>All n = 361; IG n = 181; CG n = 180<br>Proportion female at baseline:<br>0%, exclusively Boys	• Teacher training<br>• Restructured PE lessons<br>• Provision of fitness equipments<br>• HE focused on PA<br>(three times provided by research team)<br>• Additional PA sessions delivered by trained teachers<br>(90 minutes weekly)<br>• Lunch-time PA leadership sessions run by students<br>• Newsletters focused on reducing screen time for cargivers<br>• Supported by a smartphone APP<br>Intervention period: 20 weeks	WC (cm) *Boys*<br>Difference in mean change (95% CI)<br>Adjusted for baseline value, SES variables<br>IG vs CG<br>0.3 (-0.7; 1.4) p=0.549<br>Cluster design considered
Nogueria 2017[63],<br>CAPO Kids, Australia<br><br>Unit of randomisation: school	Primary schools:<br>IG n = 1; CG n = 1<br>5th and 6th grade students<br>Age (years) mean (SD):<br>All 10.6 (0.6)<br>Participants at baseline:<br>All n = 339; IG n = 185; CG n = 154<br>Proportion female at baseline:<br>No information presented	• Additional PA in units of 10 minutes<br>○ Three times a week<br>○ Based on capoeira and a combination of medium- to high-impact maneuvers for upper and lower limb loading<br>Intervention period: ~1 academic year	No effect estimate reported for WC<br>WC (cm) mean (SD)<br>*Baseline*<br>IG 67.3 (10.3); CG 66.1 (9.9)<br>*Posttest*<br>IG 68.9 (9.9); CG 70.0 (10.4)
Ochoa-Avilés 2017[64],<br>ACTIVITAL, Ecuador<br><br>schools match paired (school size, public/private, single gender/co-ed, SES variables)<br><br>Unit of randomisation: school	Middle schools:<br>IG n = 10; CG n = 10<br>8th and 9th grade students<br>Age (years) mean (SD):<br>IG 12.9 (0.8); CG 12.9 (0.8)<br>Participants at baseline:<br>All n = 1383; IG n = 691; CG n = 692<br>Proportion female at baseline:<br>IG 66.2%; CG 58.2%	• Participatory workshops with school staff and students before implementation of intervention<br>• HE focused on diet, including practical exercises<br>○ Every two weeks<br>○ Provision of interactive material<br>○ Delivered by school or external teachers<br>• Workshops on healthy diet with school food kiosk staff<br>○ Provision of supportive material<br>○ Delivered by programm staff<br>• Workshops on healthy diet for caregivers<br>○ Provision of supportive material<br>○ Delivered by programm staff<br>Intervention period: 2.5 academic year	WC (cm)<br>Difference in mean change (95% CI)<br>Adjusted for baseline variables<br>IG vs CG<br>-0.84 (-1.68; -0.28) p=0.005<br>Cluster design considered
Robinson 1999[88],<br>nn, USA<br><br>schools match paired (SES variables, scholastically)	Public elementary schools:<br>IG n = 1; CG n = 1<br>3rd to 4th grade students<br>Age (years) mean (SD):<br>IG 8.95 (0.64); CG 8.92 (0.70)<br>Participants at baseline:	• Teacher training<br>• HE focusing on screen time and PA<br>• Self-monitoring and self-reporting screen time<br>• Screen time challenge<br>• Promotion of 7h budget screen time per week	WC (cm)<br>Difference in mean change (95% CI)<br>Adjusted for age, sex, baseline value<br>IG vs CG<br>-2.3 (-3.27; -1.33) p<0.01

Unit of randomisation:<br>school	All n = 198; IG n = 95; CG n = 103<br>Proportion female at baseline:<br>IG 44.6%; CG 48.5%"	• Newsletter to caregivers/families<br>• Families were provided with electronic televison time manager<br>Intervention period: 6 months	Cluster design considered
Santos 2014[90],<br>Healthy Buddies™,<br>Canada<br><br>block randomisation (equal representation of rural and First Nations schools)<br>Unit of randomisation:<br>school	Schools with grades 1 to 6 and at least 200 students:<br>IG n = 10; CG n = 10<br>7th to 9th grade students<br>Age (years) mean (95%CI):<br>IG 9.3 (9.1; 9.5); CG 8.8 (8.6; 9.0)<br>Participants at baseline:<br>All n = 647; IG n = 340; CG n = 307<br>Proportion female at baseline:<br>IG 53.5%; CG 42.1%	• Teacher training<br>• HE focused on PA, diet, body image<br>• Peer-led-model:<br>○ Older students (9 - 12 years) as peer mentors for younger students 'buddies' (6 - 8 years)<br>• Promoting social skills<br>• Additional PA units of 30 minutes twice a week with the student pairs (mentor and buddy)<br>Intervention period: 1 academic year	WC (cm)<br>Mean change (95% CI)<br>Adjusted for age, sex, baseline value<br>IG -0.41 (-1.45; 0.64)<br>CG 1.02 (0.33; 1.71)<br>Cluster design considered
Siegrist 2013[91],<br>JuvenTUM, Germany<br><br>regional pairing of schools<br><br>Unit of randomisation:<br>school	Primary schools:<br>IG n = 4; CG n = 4<br>2nd and 3rd grade students<br>Age (years) mean (SD):<br>All 8.4 (0.7)<br>Participants at baseline:<br>All n = 724; IG n = 427; CG n = 297<br>Proportion female at baseline:<br>All 48.3%	• Teacher training<br>• Restructured PA environment, sports facilities<br>• Organized PA at recess<br>• HE lessons including short PA units and relaxation exercises<br>• Two learning sessions for parents on health related topics, especially on PA<br>• Regular (monthly) health related journals<br>Intervention period: 1 academic year	WC (cm)<br>Difference in mean change (95% CI)<br>Adjusted for age, sex, baseline value<br>IG vs CG<br>-2.7 (-2.3; -1.15) p<0.01<br>Cluster design considered
Singh 2009[92],<br>DOiT, Netherlands<br><br>stratified randomisation (urbanisation)<br>Regional pairing<br><br>Unit of randomisation:<br>school	Prevocational schools:<br>IG n = 10; CG n = 8<br>1st year Student at prevocational school<br>Age (years) mean (SD):<br>IG 12.8 (0.5); CG 12.9 (0.5)<br>Participants at baseline:<br>All n = 1108; IG n = 632; CG n = 476<br>Proportion female at baseline:<br>All 53.3%	• Teacher manual including worksheets and other material<br>• HE focused on energy balance-related behaviors and behavior change techniques<br>○ Supportive material: diary, computer-tailored advice, pedometer, additional video material<br>• Additional PE lessons (every 2nd week)<br>• Restructured assortment of school canteen<br>• Restrictied access to vending machines<br>Intervention period: 1 academic ye	WC (cm)<br>Difference in mean change (95% CI)<br>Adjusted for age, sex, baseline value<br>IG vs CG<br>*Boys*<br>-0.6 (-1.1; -0.1)<br>*Girls*<br>-0.4 (-0.8; 0.2)<br>Cluster design considered
Singhal 2006[93],<br>nn, India<br><br>Schools match paired (school size, middle SES)<br><br>Unit of randomisation:<br>school	Co-educational schools:<br>IG n = 1; CG n = 1<br>11th grade students<br>Age (years) mean (SD):<br>IG 16.04 (0.41); CG 16.0 (0.5)<br>Participants at baseline:<br>All n = 209; IG n = 101; CG n = 108<br>Proportion female at baseline:<br>IG 38.6%; CG 41.7%	HE focused on diet (weekly)<br>• Individual counselling in small group sessions by trained nutrionist (diet, lifestyle, PA)(weekly)<br>Training of volunteer students to sustain program (weekly)<br>Restructured school canteen menu<br>Monthly short counselling offer for caregivers by telephone<br>• Whole day health event at school including teachers and parents as participants	WC (cm)<br>Mean change (SD)<br>IG -0.65 (3.99)<br>CG 0.65 (4.15)<br>Difference of mean change (95% CI)<br>P=0.02 (-2.43; -0.17)

		Promotion of PA and active commuting (school information)<br>Intervention period: 6 months	
Tarro 2014[94],<br>EdAl, Spain<br><br>randomisation scheme in which schools in one city were designated to IG and schools in three other cities to CG<br><br>Unit of randomisation: town	Public and private schools:<br>IG n = 24; CG n = 14<br>7 to 8 year old students<br>Age (years) mean (SD):<br>All 8.4 (0.6)<br>Participants at baseline:<br>All n = 2350; IG n = 1550; CG n = 800<br>Proportion female at baseline:<br>All 49%	• HE provided by university trained 'Health promoting agents'<br>• Main focus on diet<br>• Each school year four units (every two weeks in two months)<br>• Provision of additional booklets (teachers) and workbooks (students) for usual classroom curriculum<br>Intervention period: 2.5 academic years	No effect estimate reported for WC<br>WC (cm)<br>Mean (95%CI)<br>*Baseline*<br>IG 59.85 (59.42; 60.28)<br>CG 59.80 (59.04; 60.57)<br>*Posttest*<br>IG 66.72 (66.2; 67.22)<br>CG 66.39 (65.39; 67.38)<br>Difference of mean change<br>IG vs CG p=0.043
Thakur 2016[68],<br>nn,<br>North India<br><br>Unit of randomisation: school	Private and public schools:<br>IG n = 2; CG n = 2<br>8th and 9th grade students<br>Age (years) mean (SD):<br>IG 13.5 (0.7); CG 13.3 (0.8)<br>Participants at baseline:<br>All n = 462; IG n = 201; CG n = 261<br>Proportion female at baseline:<br>IG 42.3%; CG 19.2%"	• HE focused on diet, PA and lifestyle disoders (e.g.obesity, hypertension)<br>○ Every 2nd week<br>○ Delivered by trained experts<br>• Diaries for students for self recording daily diet and PA<br>• Additional PA period each school day<br>• Change of menu of school canteen<br>• Dietary recommendations for caregivers<br>• Involving teachers in health assessment<br>• Parent Teachers' Association<br>*After baseline assessment: CG students were provided with information about diet and PA if wanted*<br>Intervention period: 20 weeks	WC Z-score<br>Difference in mean change (95% CI)<br>IG vs CG<br>Adjusted for sex, baseline value<br>-0.14 (-0.25; -0.03) p<0.01<br>Cluster design considered
Xu 2017[72],<br>NISOC, China<br>*Comprehensive intervention*<br>Unit of randomisation: school	Non boarding schools:<br>IG n = 15; CG n =<br>6 to 10 year old students<br>Participants at baseline:<br>All n = 7717; IG n = 3536; CG n = 3541<br>Proportion female analysed:<br>IG 49.5%; CG 49.9%	• Teacher training<br>• HE focused on diet and PA<br>• Info material for each student<br>• Supportive material: Nutrition education Handbook<br>• Periodical evaluation of menu in school lunch cafeteria with recommendations for improvement<br>• Additional PA sessions for 10 min,<br>○ Delivered by classroom teachers<br>○ Twice every school day<br>• HE for caregivers (twice)<br>Intervention period: 1 academic year	WC (cm)<br>Regression Coefficient β (95% CI)<br>Adjusted for age, sex, baseline value, other confounding factors<br>IG vs CG<br>-0.5 (-0.6; -0.3)<br>*regression coefficient corresponds to the difference between CG and IG*<br>Cluster design considered

CG = control group, CI = Confidence Interval, Diff = difference, FU = follow-up, HE = health education, IG = intervention group, IQR = interquartile range, MVPA = moderate to vigorous physical activity, nn = no name, PA = physical activity, PE = physical education, RCT = Randomized Controlled Trial, SD = standard deviation, SE = standard error, SES = socio economic standard, vs = versus, WC = waist circumference
