# Supplementary material for: Waist circumference as a parameter in school-based interventions to prevent overweight and obesity - a systematic review and meta-analysis
Source: BMC Public Health. 2024 Oct 17;24:2864. doi: 10.1186/s12889-024-20354-7 (PMC11488270; doi:10.1186/s12889-024-20354-7)
Supplement: Supplementary file 3 — Supplementary Material 3: Additional file 3. Design and characteristics – non-randomised controlled trials: included studies sorted alphabetically. [file 12889_2024_20354_MOESM3_ESM.pdf]

Kula et al. Additional file 3. Design and characteristics – non-randomised controlled trials: included studies sorted alphabetically

**Included studies sorted alphabetically: Non-randomised controlled trials**			
First author & publication year, program name, country of origin, study design specifics	Sample / Participants	Key components of intervention	Main results regarding waist circumference
Adab 2014[74],<br>BEACHeS, UK<br><br>schools were matched (school size, SES variables, geographical location)<br><br>Cluster level: school	Primary schools in Birmingham with ≥ 50% of pupils from South Asian background:<br>IG n = 4; CG n = 4<br>1st and 2thnd grade students<br>Age (years) mean (SD):<br>IG 6.53 (0.59); CG 6.44 (0.58)<br>Participants at baseline:<br>All n = 574; IG n = 269; CG n = 305<br>Proportion female at baseline:<br>IG 46.5%; CG 50.2%"	• Teacher / school staff training<br>• Promotion of PA within everyday school life (short sessions within classes, organised break times activities, environmental restructuring, Incentive scheme)<br>• Promotion of development of a healthy lifestyle ('Villa Vitality day' with Footballclub coaches and interactive session on healthy eating and healthy lifestyle, weekly lessons in class over six weeks)<br>• Cooking courses for family members over five weeks<br>• Information for families on local leisure opportunities including tryouts<br>• Training of volunteers to initiate community walking programs<br>Intervention period: 1 academic year	WC (cm)<br><br>Difference in mean change (95% CI)<br>Adjusted for age, sex, baseline value, potential confounders<br>IG vs CG<br>-0.86 (-1.87; 0.15)<br>p=0.09<br>Cluster design considered
Centis 2012[76],<br>nn, Italy<br><br>IG and CG were matched by SES variables<br><br>Cluster level: school	Primary schools:<br>All n = 7<br>4th grade students<br>Age (years) mean (SD):<br>IG 9.33 (0.29); CG 9.36 (0.35)<br>Participants at baseline:<br>All n = 209; IG n = 103; CG n = 106<br>Proportion female at baseline:<br>All 52.2%; IG 51.5% ; CG 52.8%	*Before start of Intervention:*<br>Meeting with teachers and parents *in CG and IG*<br>importance of healthy nutrition and of regular PA<br>adjustment in the daily supply of food at school if needed<br><br>• HE on PA by university experts (3 times)<br>• Restructured PE lessons by university experts<br>• Step counters for motivational support<br>• One event on healthy nutrition by physician and expert on motivational acitivities (theoretical input and practical part)<br>• Motivational meetings with caregivers focusing on the benefits of healthy diet and PA by teachers and nutrition experts (weekly in the first 4 months)<br>Intervention period: 1 academic year	No effect estimate reported for WC<br><br>WC (cm)<br><br>Mean change (SD)<br>IG 1.5 (3.0); CG 2.3 (3.2)<br>Difference in mean change<br>IG vs CG<br>p=0.59
Contardo Ayala 2018[52],<br>nn, Australia<br><br>students matched on year level and school subject	Public secondary school: n= 1<br>IG = Timetabled classes in intervention classroom<br>7th, 10th and 11th grade students<br>Age (years) mean (SD):	• Height adjustable desks<br>• Breaking up sitting time regularly (every 15 min)<br>• Additional supportive prompts<br>• Additional posters on health impacts of sitting time, tips and strategies on how to reduce classroom sitting time	WC (cm)<br>Difference in mean change (95% CI)<br>Adjusted for sex, baseline value<br>IG vs CG<br>-2.6 (-5.0; -0.3)

Cluster level: class	All 14.8 (1.7), range 12-17 years<br>Participants at baseline:<br>All n = 88; IG n = 49; CG n = 39<br>Proportion female at baseline:<br>IG 38.8%; CG 48.7%	• Teacher instruction and printed manual"<br>Intervention period: 5 months	Cluster design considered
Eyre 2016[54],<br>nn, UK<br><br>Cluster level: not applicable	Primary school n = 1<br>students from deprived South Asian family background<br>IG: 4th to 5th grade students<br>CG: 6th grade students<br>Age (years) mean (SD):<br>IG 9.48 (0.62); CG 11.12 (0.32)<br>Participants at baseline:<br>All n = 122; IG n = 89; CG n = 33<br>Proportion female at baseline:<br>No information presented	• Pedometer challenge as virtual walk<br>• HE on diet, PA and other health related topics (usual science class weekly)<br>• HE in last intervention week<br>• (integrated health topic in every curriculum lesson)<br>• Promotion of skipping (short instruction and provision with skipping ropes for each child)<br>• Additional afterschool activity sessions (four times, weekly)<br>Intervention period: 6 weeks	WC (cm)<br>Mean change (SD)<br>IG -1.73 (4.48); CG -0.21 (3.49)<br>Difference in mean change<br>IG vs CG (95 %CI)<br>p=0.001 (-3.4; 0.36)<br>Cohen's *d* 0.44<br><br>WC SD-score<br>Mean change (SD)<br>IG -0.79 (0.99); CG -0.11 (0.71)<br>Difference in mean change<br>IG vs CG (95 %CI)<br>p=0.001 (-1.21; 0.29)<br>Cohen's *d* 0.96
Feng 2016[57],<br>nn, USA<br><br>IG and CG matched on school-level and demographic characteristics<br><br>Main target group:<br>Disadvantaged Hispanic children<br><br>Cluster level: school/class	Sample schools with high proportion of Student from low SES and Hispanic family background<br>Kindergarten to 2nd grade students<br>Age (years) mean (SD):<br>All 6.68 (0.96), range 5-9 years<br>Participants at baseline:<br>All n = 555<br>Proportion female at baseline:<br>All 53%	• Teacher training<br>• HE on healthy eating<br>(10 units plus take-home workbooks)<br>• Restructured PE lessons<br>• HE on PA by take-home workbooks<br>• Monthly newsletters to parents plus information sheets on PA<br>• Family fun nights twice a year<br>*Special component for children with overweight (> 30% of participants IG, ~40% of eligible families took part)*<br>Intervention period: 1,5 academic year	No effect estimate reported for WC<br><br>WC (cm)<br>Mean change (SD)<br>*Baseline*<br>IG 59.81 (9.29); CG 58.87 (9.56)<br>*Posttest*<br>IG 64.12 (11.48); CG 65.38 (12.80)
Hatzis 2010[81],<br>Cretan Health and Nutrition Education Program, Greece<br><br>Cluster level: counties	Primary schools:<br>IG n = 24; CG n = 16<br>1st grade students<br>Age (years) mean (SD):<br>All 6.3 (0.4)<br>Participants at baseline:<br>All n = 1046; IG n = 602; CG n = 444	• Teacher training<br>• HE focusing on diet, PA and fitness, including behavior change methods and motivational units (about 15 units per academic year)<br>• Workbooks for grade 1-3 focusing on diet, PA and fitness, also including other general health issues as dental hygiene and accident prevention<br>• Restructured PE lessond including a theoretical part on goal	No effect estimate reported for WC<br><br>WC (cm)<br>Adjusted for sex, baseline value, parental education<br>Difference in mean change<br>p>0.05<br>Cluster design considered

	Proportion female at baseline:<br>All 47.9%	setting, self-improvement and aspects of healthy PA (twice weekly)<br>• Meetings and information for caregivers (twice a year)<br>*CG: screening results plus brief comment per mail to parents*<br>Intervention period: 6 academic years	
Kain 2009[83],<br>nn, Chile<br><br>IG and CG schools with similar SES characteristics<br><br>Cluster level: school	Primary schools:<br>IG n = 3; CG n = 1<br>7th grade students<br>Age (years) mean (SD):<br>IG 10.0 (2.3); CG 9.9 (2.1)<br>Participants at baseline:<br>All n = 2430; IG n = 1759; CG n = 671<br>Proportion female at baseline:<br>IG 48.9%; CG 39.3%	• Teacher training<br>• HE on healthy eating by trained teachers<br>• 90 minutes additional PE classess weekly by trained teachers<br>• Active recess in first intervention period<br>• HE for caregivers by Nutrionists (twice)<br>*Special component: Group meeting by nutrionist advising caregivers of children with obesity*<br>Intervention period: 1,5 academic years	No effect estimate reported for WC<br><br>WC (cm)<br>Mean change (SD)<br>*Boys*<br>*Baseline*<br>IG 64.9 (9.7); CG 65.6 (10.6)<br>*Posttest*<br>IG 68.0 (8.8); CG 68.5 (9.4)<br>*Girls*<br>*Baseline*<br>IG 64.9 (9.9); CG 67.7 (9.1)<br>*Posttest*<br>IG 64.0 (10.2); CG 67.3 (9.1)<br><br>No statistical significant effect reported
Ostrowski 2018[65],<br>ROAD, USA<br><br>Cluster level: not applicable	Middle schools:<br>No information presented<br>CG: 6th to 8th grade students year 1<br>IG: 6th to 8th grade students year 2<br>Age (years) mean (SD):<br>All 12.7 (0.9)<br>Participants at baseline:<br>All n = 791; IG n = 469; CG n = 322<br>Proportion female at baseline:<br>All 53.0 %; IG 54.4 %; CG 52.5%	• HE focused on diet and PA<br>○ 12 sessions delivered by program team)<br>○ Targeting home environment (6th graders), school environment (7th graders), community environment (8th graders)<br>• Voluntary additional PA sessions (three times a week)<br>Intervention period: 4 months	WC (cm)<br><br>Mean change (cm)<br>IG -0.16<br>CG -0.01<br>Adjusted for age, sex, baseline value<br>IG vs CG<br>p>0.05
Pérez-Solis 2015[66],<br>Tú decides tu salud. ¡Ponte a vivir!, Spain<br><br>schools of similar socioeconomic characteristics<br><br>Cluster level: school	Public schools:<br>IG n = 1; CG n = 1<br>1st to 5th grade students<br>Age (years) mean (SD):<br>IG 8.19 (1.5); CG 8.5 (1.46)<br>Participants at baseline:<br>All n = 340; IG n = 120; CG n = 220	• HE focused on diet<br>• Workshop on healthy nutrition for students<br>○ Once per term, delivered by paediatricians from research team<br>• Classroom acitvities on same subjects every two weeks or monthly<br>• Educational talk for caregivers on same subjects	No effect estimate reported for WC<br><br>proportion of individuals with a WC >95th percentile<br>*Baseline*<br>IG 55.0% CG 54.8%<br>*Posttest*<br>IG 57.5% CG 56.1%

	Proportion female at baseline:<br>IG 45.0%; CG 49.1%	○ Once per term, delivered by paediatricians from research team<br>• Monthly meetings with teachers to study healthy lifestyle habits<br>• Joint 2-h long workshop on healthy diet (paediatricians, teachers, caregivers; once each academic year)<br>• Information material for caregivers focused on PA, sedentary time and diet<br>• Promotion of PA at recess<br>• Ban of game consoles and mobile phones during school hours<br>Intervention period: 2 academic years	
Ronsley 2014[89],<br>Healthy Buddies™, Canada<br><br>First Nations<br><br>Cluster level: school	First Nation schools in remote communities:<br>IG n = 2; CG n = 1<br>Kindergarden to 12th grade students<br>Age (years) mean (SD):<br>IG 10.9 (3.5); CG 10.6 (3.4)<br>Participants at baseline:<br>All n = 179; IG n = 118; CG n = 61<br>Proportion female at baseline:<br>IG 48.3%; CG 49.2%	• Teacher training<br>• HE focused on PA, diet, body image<br>• Peer-led-model:<br>○ Older students (9 - 12 years) as peer mentors for younger students 'buddies' (6 - 8 years)<br>• Promoting social skills<br>• Additional PA units of 30 minutes twice a week with the student pairs (mentor and buddy)<br>*Original Healthy Buddies program was cultural adapted*<br>Intervention period: 1 academic year	WC (cm)<br>Mean (SD)<br>*Baseline*<br>IG 77.1 (18.8) CG 75.8 (16.6)<br>*Posttest*<br>IG 75.0 (17.1) CG 75.5 (15.3)<br>Difference in mean change<br>Adjusted for age, sex, baseline value<br>IG vs CG<br>p=0.154
Vieira 2018[69],<br>PHS pro, Portugal<br><br>Cluster level: school	Elementary schools:<br>IG n = 1; CG n =3<br>6th grade students<br>Age (years) mean (SD):<br>All 11.2 (0.6)<br>Participants analyzed:<br>All n = 449; IG n = 219; CG n = 230<br>Proportion female at baseline:<br>No information presented	HE focused on diet and PA (one unit per month)<br>Intervention period: 1 academic year	No effect estimate reported for WC<br><br>WC (cm)<br>Mean change (SD)<br>IG -0.3745 (2.1805)<br>CG 0.297 (2.9844)<br>Difference in mean change<br>p=0.015
Wadolowska 2019[70],<br>ABC of Healthy Eating, Poland<br><br>Cluster level: class	Secondary schools:<br>Sample classes:<br>IG n = 32; CG n = 16<br>11 to 12 years old students<br>Age (years) mean (95%CI):<br>All 11.9 (11.9; 12.0) Participants at baseline:<br>All n = 464; IG n = 319; CG n = 145	• HE focused on diet and PA<br>○ Talks and workshops<br>○ Interacitve seminars<br>○ Delivered by researchers<br>○ Teachers were present during educational activities<br>Intervention period: 3 weeks	WC Z-score<br>Mean change (95% CI)<br>IG -0.05 (-0.12; 0.02)<br>CG 0.08 (0.02; 0.14)<br>Difference in mean change<br>Adjusted for baseline value<br>IG vs CG<br>-0.13 p<0.05

	Proportion female at baseline:<br>All 53.4%; IG 55.8%; CG48.3%		
Weston 2016[71],<br>FFAB, UK<br><br>in each study arm: one school with high and with low SES characteristics<br><br>Cluster level: school	Secondary schools:<br>IG n = 2; CG n = 2<br>9th grade students<br>Age (years) mean (SD):<br>IG 14.1 (0.3); CG 14.1 (0.3)<br>Participants at baseline:<br>All n = 101; IG n = 41; CG n = 60<br>Proportion female at baseline:<br>IG 24.2%; CG 50%"	• Partly restructured PE lessons (High Intensitivity Interval Training - HIT)<br>• Additional voluntary HIT session at lunch break or after school (weekly)<br>• All HIT sessions delivered by researcher<br>• Intensitiy increased over time<br>• Motivational award<br>Intervention period: 13 weeks	WC (cm)<br><br>Difference between mean posttest values (95 %CI)<br>Adjusted for sex, baseline value, maturity offset<br>IG vs CG<br>-3.9 (-6.1; -1.6)
Yang 2016[73],<br>nn, South Korea<br><br>schools matched according to grades<br><br>Cluster level: school	Elementary and middle schools:<br>IG n = 3; CG n = 2<br>4th and 7th grade students<br>Age (years) mean (SD):<br>IG 10.9 (1.6); CG 11.0 (1.5)<br>Participants at baseline:<br>All n = 768; IG n = 418; CG n = 350<br>Proportion female at baseline:<br>IG 26.8%; CG 20.9%	• HE focused on diet and PA<br>○ 5-10 minute units each day<br>○ During rest or lunch time<br>○ Per TV screen in each classroom<br>• Design materials to encourage PA at staircases and hallway (paintings etc.)<br>• According to baseline results, indivdual recommendations were provided in form of a printed handout including results, personalized suggestions and recommended dily calorie requirements<br>*Additional component for Student with overweight/obesity and their caregivers over 12 week summer vacation*<br>Intervention period: 1 academic year	WC (cm)<br><br>Difference in mean change (95% CI)<br>Adjusted for age, sex, baseline value<br>IG vs CG<br>2.5 (1.9; 3.2) p<0.001<br>Cluster design considered
CG = control group, CI = Confidence Interval, FU = follow-up, HE = health education, IG = intervention group, nn = no name, PA = physical activity, PE = physical education, SD = standard deviation, vs = versus, WC = waist circumference			
